# Inhibition of IDH3α Enhanced the Efficacy of Chemoimmunotherapy by Regulating Acidic Tumor Microenvironments

**DOI:** 10.3390/cancers15061802

**Published:** 2023-03-16

**Authors:** Lingling Zhang, Yang Song, Xiaoyan Dai, Wenwen Xu, Mengxia Li, Yuxi Zhu

**Affiliations:** 1 Department of Oncology, The First Affiliated Hospital of Chongqing Medical University, Chongqing 400016, China; 2 Cancer Center, Daping Hospital, Army Medical University, Chongqing 400042, China; 3 Department of Oncology, Jinshan Hospital of the First Affiliated Hospital of Chongqing Medical University, Chongqing 400016, China

**Keywords:** cancer, IDH3α, chemoimmunotherapy, cGAS-STING, acidic tumor microenvironment

## Abstract

**Simple Summary:**

Isocitrate dehydrogenase 3α (IDH3α) is highly expressed in many cancers and is associated with poor patient prognosis. However, it is not clear whether the aberrant expression of IDH3α is related to the efficacy of chemoimmunotherapy against cancer. We used in vivo and in vitro experiments and bioinformatics to explore the potential role of IDH3α in chemoimmunotherapy treatment. Our results indicate that IDH3α overexpression leads to resistance to chemoimmunotherapy by regulating the acidic tumor microenvironments and the cGAS–STING pathway. Our results provide initial preclinical evidence that the combination of IDH3α knockdown and chemoimmunotherapy may improve the therapy for cancer patients.

**Abstract:**

In recent years, chemoimmunotherapy has become effective in some advanced cancers, but its effect is still limited. Transcriptional upregulation of isocitrate dehydrogenase 3α (IDH3α) can promote tumor initiation and progression. However, it is not clear whether the aberrant expression of IDH3α is related to the efficacy of chemoimmunotherapy in cancers. Here, we found that IDH3α was elevated in uterine cervical cancer (UCC) and lung adenocarcinoma (LUAD) samples by using public databases. High expression of IDH3α could promote the epithelial–mesenchymal transition (EMT), alter the intracellular redox status, promote glycolysis, and induce an acidic microenvironments in cancer cells. Furthermore, we found that inhibition of IDH3α combined with chemoimmunotherapy (cisplatin and programmed cell death ligand 1 (PD-L1) antibodies) activated the cGAS–STING pathway, promoted CD8^+^ T cell infiltration, and decreased tumor growth in mouse models of cervical cancer. In conclusion, our data indicate that silencing IDH3α sensitizes tumors to chemoimmunotherapy by modulating the acidic microenvironment and activating the cGAS–STING pathway.

## 1. Introduction

Cisplatin (CDDP)-based chemotherapy is a standard treatment for many cancers [1]. However, resistance to chemotherapy is associated with poor survival [2]. Recently, immune checkpoint inhibitors (ICIs) targeting the programmed cell death 1 (PD-1)–programmed cell death ligand 1 (PD-L1) axis have emerged as a novel and effective strategy for non-small cell lung cancer (NSCLC) [3] and uterine cervical cancer (UCC) [4]. In addition, the combination of ICIs and chemotherapy has been widely used in cancer patients. However, the effect of this combination is still limited. Some studies have found that conventional chemotherapy can reduce immune function, such as the number of CD4^+^ and CD8^+^ T lymphocytes, which may limit the use of ICIs in combination therapy [5,6].

Energy reprogramming is considered a hallmark of cancer, because it promotes cancer initiation and cancer cell growth [7]. In addition, increasing evidence suggests that cancer metabolism may influence the immune response and induce resistance to ICIs by regulating the acidic tumor microenvironments and affecting the expression of immune molecules [8]. The isocitrate dehydrogenase (IDH) family is one of the key families of enzymes in the tricarboxylic acid cycle that catalyze the conversion of isocitrate to α-ketoglutarate (α-KG). IDH1/2 missense mutations alter enzyme catalysis and lead to the production of the tumor metabolite 2-hydroxyglutarate (2-HG), which acts as a competitive inhibitor of α-KG [9]. Furthermore, the aberrant expression of isocitrate dehydrogenase 3α (IDH3α) can regulate the proliferation, invasion, and migration of various tumors [10], and the overexpression of IDH3α correlates with poor prognosis in lung and breast cancer [11].

On the other hand, the cGAS/STING pathway regulates both innate and adaptive immunity. When cGAS senses tumor-derived double-stranded DNA, it activates STING by producing cGAMP. The activation of the STING pathway induces anti-tumor immune responses by activating multiple pathways, including IRF3 and NF-κB [12,13], and then remodeling the inhibitory TME and the sensitizing the ICI by regulating the level of other immune and inflammatory factors. In several tumor models, the activation of STING in non-cancer cells in the tumor microenvironment, usually dendritic cells or macrophages [14,15], is necessary to trigger an anti-tumor immune response. Unlike the activation of STING in non-cancer cells, the role of STING in anti-cancer immunity in cancer cells is less studied. It has been reported that 5-fluorouracil (5-FU) reduces tumor load by activating the STING pathway in the tumor [16]. In the absence of the intrinsic STING of cancer cells, a higher dose of 5-FU was required to reduce the tumor load. 5-FU treatment leads to an increase in T cells in the tumor, and the depletion of T cells significantly reduced the efficacy of 5-FU in vivo [16]. Therefore, the inhibition of the cGAS–STING pathway in tumor cells may induce resistance to chemoimmunotherapy.

In this study, we focused on the relationship between the overexpression of IDH3α and the acidic tumor microenvironments and confirmed whether the inhibition of IDH3α could sensitize tumors to chemoimmunotherapy by activating the cGAS–STING pathway. Therefore, we may provide a new potential therapeutic target against tumors for patients in clinical practice.

## 2. Materials and Methods

### 2.1. Bioinformatics Analysis

CBioPortal (https://www.cbioportal.org/, accessed on 12 November 2021), Oncomine (http://www.oncomine.org/, accessed on 7 November 2021) and HPA (http://www.proteinaltas.org/, accessed on 2 January 2022) databases were used to analyze the expression of IDH3α in UCC, lung cancer, and non-cancerous tissues. Patients’ survival data were obtained from TCGA and GEO (GSE13213) to perform Kaplan–Meier analysis. KEGG and GO enrichment and immune cell infiltration analysis were previously described [17].

### 2.2. Cell Line Culture and Drug Treatment

HeLa (RRID: CVCL 0030), CaSki (RRID: CVCL 1100), SiHa (RRID: CVCL 0032), U14 (RRID: CVCL 9U56), and NCI-H1299 (RRID: CVCL 0060) cancer cells were purchased from the Cell Bank of Chinese Academy of Sciences (Shanghai, China) and authenticated by the supplier (Shanghai, China). SiHa and U14 were cultured in DMEM (Gibco-BRL, Karlsruhe, Germany), and the others were cultured in the RPMI1640 (Gibco-BRL, Karlsruhe, Germany) medium, supplemented with 10% fetal bovine serum (Gibco, New York, NY, USA) and 1% penicillin–streptomycin (Gibco, New York, NY, USA) at 37 °C in a humidified atmosphere containing 5% CO_2_. Culture media containing different concentrations of cisplatin-treated cells were incubated for 24 h. STING inhibitor C-176 (S6575, Selleck), at 20 µM, was used to treat cells for 24 h.

### 2.3. Construction of Stable Cell Lines

UCC and LUAD cells (1 × 10^5^) were infected at a multiplicity of infection (MOI) of 20 with scrambled-shRNA, *IDH3A*-shRNA, vector, or *IDH3A*-overexpressing lentiviruses (GeneChem, Shanghai, China) for 48 h at 37 °C in the presence of 5 μg/mL of polybrene. The expression of IDH3α in transduced cells was validated by qPCR and WB.

### 2.4. CCK8 Assay and EdU Assay

For the CCK8 assay, we seeded 3000 cells/well in 96-well plates. After 24, 48, 72, and 96 h, CCK-8 at 10 μL/well (Dojindo, Kumamoto, Japan) was added for incubation for 2 h, and the absorbance was measured at 450 nm with a microplate reader (Molecular Devices). For the EdU incorporation assay, we used the EdU Incorporation Assay Kit (R11053.9, Ribobio, Guangzhou, China), following the manufacturer’s protocol. Images were taken in five randomized fields under a fluorescence microscope.

### 2.5. Colony Formation Assays

For the colony formation assay, 500 cells per well were seeded into 6-well plates, with IDH3α knockdown or a negative control. After 10–14 days, 4% paraformaldehyde was used to fix the cells for 20 min, and crystal violet was used to stain the colonies for 20 min at room temperature. The colony formation rate was calculated using the following formula: colony formation rate = number of colonies/number of seeded cells × 100.

### 2.6. Cell Invasion Assay and Wound Healing Assay

For invasion analysis, 5 × 10^4^ cells in 200 μL of serum-free medium were seeded into 8 mm pore Transwell inserts (3422, Corning, NY, USA), coated with 300 μg/mL of Matrigel (356234, Corning), and in the lower chamber contained 600 µL of RPMI containing 30% FBS. After a 24 h incubation, the number of cells that adhered to the subsurface of the membrane was counted under a microscope. For the wound healing assay, confluent cells were scraped with a 200 μL pipette tip to create an artificial wound and incubated with fresh medium. The distance migrated was quantified by taking images at 0 and 24 h. This assay was repeated thrice.

### 2.7. Flow Cytometry

Flow cytometric analysis of the cell cycle and apoptosis was conducted as previously described [18]. For apoptosis, cells were collected, washed twice with PBS, stained with DAPI (PB450) and annexin V (APC), and analyzed with flow cytometry. To assess the cell cycle, cells were fixed in ice-cold 70% ethanol for 24 h and analyzed using flow cytometry (BD Biosciences, San Jose, CA, USA). MitoTracker Red CMXROS (C1035, Beyotime, Shanghai, China) was used to examine the mitochondrial content according to the manufacturer’s instructions and analyzed by flow cytometry.

### 2.8. RNA-seq

Total RNA was isolated from the control and CaSki-shIDH3α cells (three biological replicates). RNA quality control was performed using the Agilent 2100 Bioanalyzer. RNA-seq libraries were generated using the NEBNext Ultra RNA Library Prep Kit for Illumina by following Illumina protocols. Libraries were quantified using an Agilent 2100 Bioanalyzer, and the pooled libraries were sequenced with an Illumina HiSeq 4000 system using Illumina reagents and protocols Novogene (Beijing, China).

### 2.9. qPCR

Total RNA was extracted from cultured cells and mouse tumor models using the Simply P Total RNA Extraction Kit (Bio Flux, Tokyo, Japan), according to the manufacturer’s instructions. Reverse transcription was performed according to the manufacturer’s instructions (Takara Biotechnology, Dalian, China). The qPCR reaction was performed using an SYBR Green PCR Kit (Takara, Biotechnology, Japan) with primers (Table S1), and the real-time PCR system (CFX96 System, Bio-Rad) was used to amplify samples under the following conditions: 95 °C for 30 s, followed by 39 cycles of 95 °C for 5 s and 60 °C for 30 s. Gene expression levels were normalized to β-actin mRNA levels and expressed as the relative fold change in expression compared with the control.

### 2.10. Western Blotting

After culture and treatments, cells were harvested and lysed in protein extraction reagent (78501, ThermoFisher) and mixed with 1% protease inhibitor mixture at 4 °C for 30 min. Protein samples (40 μg) were separated by SDS-PAGE and transferred to PVDF membranes. Next, the membranes were blocked with 10% non-fat milk for 1 h and incubated overnight at 4 °C with primary antibodies: anti-IDH3α (15909-1-AP, Proteintech, Chicago, IL, USA), anti-HIF-1α (ab51608, Abcam, Cambridge, UK), anti-E-cadherin (60335-1-Ig, Proteintech), anti-N-cadherin (66219-1-Ig, Proteintech), anti-Vimentin (ab8978, Abcam), anti-slug (12129-1-AP, Proteintech), anti-cGAS (15102, CST, Danvers, MA, USA), anti-sting (13647, CST), anti-phospho-sting (Ser366) (19781,CST), anti-TBK1 (38066, CST), anti-phospho-TBK1 (S172) (ab109272, Abcam), anti-IRF3(ab76409, Abcam), anti-phospho-IRF3(S386)(ab76493, Abcam), anti-p65 (ab32536, Abcam), anti-phospho-p65 (S536) (ab76302, Abcam), anti-PD-L1 (ab213524, Abcam), and β-tubulin (sc-8432, Proteintech). Finally, the membranes were incubated with HRP-conjugated secondary antibodies at room temperature for 1 h. Western blotting analysis was performed using a Bio-Rad chemiluminescence imager. Original blots can be found at Supplementary Figure S5.

### 2.11. Quantification of Glucose Uptake, L-lactate, NAD+/NADH, and ROS

BiVision’s colorimetric assay was used for glucose uptake (K676-100) quantification, and Abcam’s Colorimetric Kit was used for the quantification of L-lactate (ab65331), ROS (ab186027), and NAD+/NADH (ab65348) levels.

### 2.12. Chemosensitivity Assay

To test the influence of IDH3α on the sensitivity of cervical cancer cells to cisplatin, 4 × 10^3^ HeLa (control, shIDH3α) and CaSki (Control, shIDH3α) cells were seeded in 96-well plates. Culture media containing different concentrations of cisplatin were added after HeLa cell adherence. CaSki cell viability was measured at 0, 24, 48, 72, and 96 h using the CCK-8 assay, following the manufacturer’s instructions, after 10 μM cisplatin treatment. The inhibition rate was calculated based on the absorbance at 450 nm.

### 2.13. In Vivo Experiments

Six-week-old female C57BL/6J wild-type mice were used to generate experimental tumors by grafting a mouse cervical carcinoma cell line (U14). Mice were injected in the groin, according to the experiment, with 5 × 10^6^ U14-control cells and U14-shIDH3α cells. After 7 days, the animals harboring 2 kinds of tumor models were randomized into 3 groups and received intraperitoneal injections of vehicle (PBS), cisplatin (5 mg/kg), and/or anti-PD-L1 (Bio X cell^®^, 100 μg/mouse), respectively. Antibodies were injected at a dose of 100 μg/mouse at days 2, 5, 8, and 12. Cisplatin was injected at a dose of 5 mg/kg on days 1, 3, 5, 7, and 10 (shIDH3α + CDDP group) and 1, 4, 7, and 11 (shIDH3α + CDDP + PD-L1 group). The tumor volume was measured every 3 days using the following formula: L × W^2^/2. The animal experiments were approved by the Ethics Committee of the First Affiliated Hospital of Chongqing Medical University.

### 2.14. HE and Immunohistochemistry Assays

Tumor tissues were prepared as 3 μm thick paraffin sections. The slides were stained with hematoxylin, washed, and counterstained with eosin (HE). For IHC staining, the slides were dewaxed with xylene and rehydrated using graded ethanol. Autoclaved antigen retrieval was performed in sodium citrate HCl buffer, an endogenous H_2_O_2_ enzyme blocker (MXB, SP KIT-A3) was used to block the tissues for 10 min, and 5% goat serum was used for blocking at 37 °C for 1 h. The sections were incubated at 4 °C overnight with primary antibodies: anti-IDH3α rabbit antibody (1:200, 15909-1-AP, Proteintech), anti-Ki67 rabbit antibody (1:200, ab16667, Abcam), CD45 (1:1200, GB11066, Servicebio, Wuhan, China), CD4 (1:200, GB13064-2, Servicebio), and CD8 (1:200, GB13429, Servicebio). The HRP-conjugated secondary antibody was incubated with the sections for 30 min at 37 °C. The sections were rinsed with PBS, developed with diaminobenzidine substrate, and counterstained with hematoxylin for nuclear staining. A light microscope was used to observe and capture images of the histopathological changes. Staining was blindly scored by a clinical pathologist. According to the percentage of IDH3α or Ki67-positive cells in the total number of cancer cells, the evaluable sections were classified into four IHC scores: IHC score 0, 0% positive; IHC score 1, 1–10% positive; IHC score 2, 11–50% positive; and IHC score 3, >50% positive.

### 2.15. Immunofluorescence

The excised tumor tissue was fixed in 4% paraformaldehyde and embedded in paraffin. The paraffin blocks were sectioned, deparaffinized, and microwaved in EDTA antigen retrieval buffer (pH 8.0) for 20 min. They were incubated overnight at 4 °C with anti-mouse CD45 antibody, and then the corresponding HRP-labeled goat anti-rabbit IgG was used for incubation for 50 min at room temperature the next day. TSA (1:300, G1223, Servicebio) was added, and microwaves were used to remove the primary and secondary antibodies bound to the tissue. Next, the anti-mouse CD8 antibody was added and incubated with the sections overnight at 4 °C, and the corresponding fluorescently labeled goat anti-rabbit IgG was added and incubated with the sections at room temperature for 50 min in the dark. DAPI (G1012, Servicebio) was incubated at room temperature for 10 min in the dark to counterstain nuclei. Finally, data were collected using a fluorescence microscope.

### 2.16. Statistical Analysis

All the data are presented as the mean (±SD). One-way ANOVA was used for single comparisons with >2 groups. Two-way ANOVA was used for multiple comparisons within the groups. Statistical analyses were performed using GraphPad Prism V8.0 (GraphPad Software, San Diego, CA, USA), and * (*p* < 0.05) indicates statistically significant difference.

## 3. Results

### 3.1. Upregulation of IDH3α Is Associated with Poor Patient Survival in UCC and LUAD

**Figure 1:**
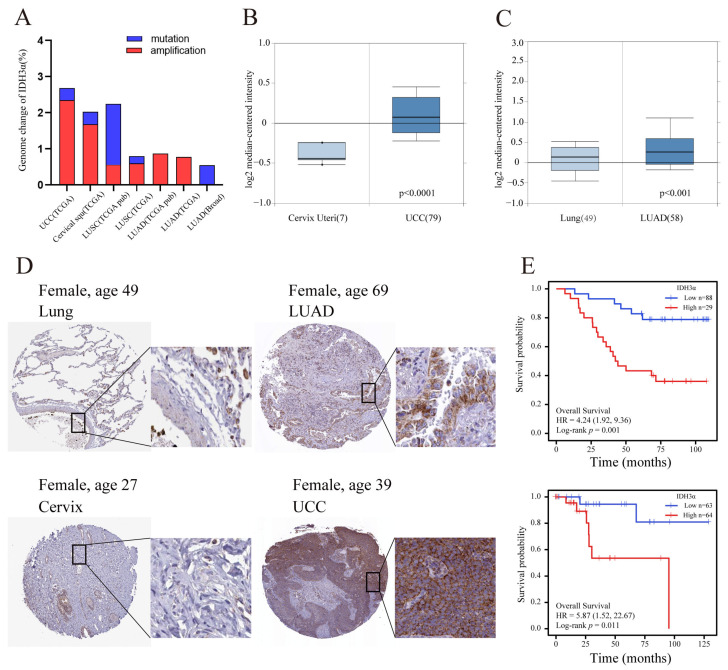
The expression level of IDH3α and prognostic analysis in UCC and LUAD. (**A**) Genomic amplification and mutation of IDH3α in UCC and lung cancer (data from TCGA). (**B**) The expression of IDH3α in cervical cancer and normal cervix tissues was analyzed by using the Oncomine database (T > N). (**C**) The expression of IDH3α in LUAD and normal lung tissues was analyzed by using the Oncomine database (T > N). (**D**) IHC staining of IDH3α in lung cancer and normal lung tissue (upper), as well as cervical cancer and normal cervical tissue (lower), in the HPA database. (**E**) The KM method was used to establish survival curves, and the log-rank test was used to compare survival curves across subgroups. (Upper) LUAD, (lower) UCC. Data are shown as mean ± SD.

Previous studies have reported that IDH3α is up-regulated in many cancers [11,19]. In the cBioPortal database, we found that amplification was the major genomic change in IDH3α in UCC and lung cancer (Figure 1A). By using the Oncomine database, we confirmed that the expression of IDH3α in UCC and LUAD tissues was significantly higher than that in non-cancerous tissues (Figure 1B,C). In addition, compared to normal tissues in the HPA database, the protein expression levels of IDH3α were higher in uterine cervical and lung cancer tissues (Figure 1D). High expression levels of IDH3α were associated with poorer overall survival in UCC and LUAD patients (Figure 1E).

### 3.2. Downregulation of IDH3α Exhibited Tumor-Suppressive Functions in Cancer Cell Lines

**Figure 2:**
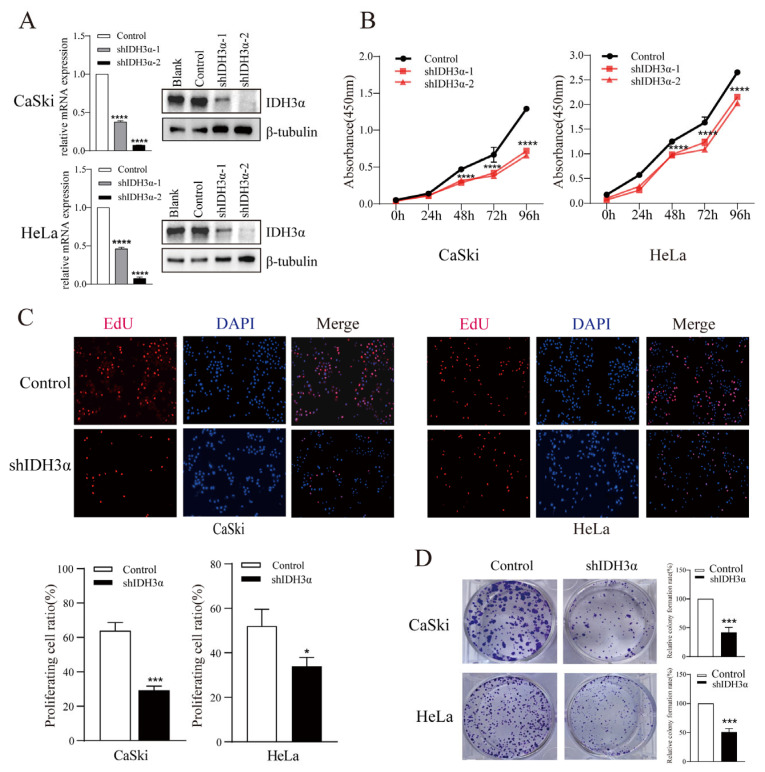
The effects of IDH3α knockdown on proliferation, apoptosis, and cell cycle distribution in multiple cancer cell lines. (**A**) IDH3α-knockdown CaSki and HeLa cells were confirmed using qPCR and WB. (**B**,**C**) CCK-8 assay (**B**) and EdU assay (**C**) showed that the silencing of IDH3α expression suppressed cell vitality. (**D**) The silencing of IDH3α suppressed colony-forming ability in UCC cells. Data are shown as mean ± SD. * *p* < 0.05, *** *p* < 0.001, and **** *p* < 0.0001.

To elucidate the biological function of IDH3α in cancers, we performed a series of cell experiments. The downregulation of IDH3α expression in HeLa and CaSki cells was confirmed by qPCR and WB (Figure 2A). Using the CCK-8 assay, we found that the downregulation of IDH3α expression significantly decreased the viability of UCC cells (Figure 2B). The EdU assay revealed that the number of shIDH3α cells in the proliferative phase was significantly reduced (Figure 2C). In the colony formation assay, the quantity of colonies of HeLa and CaSki cells was significantly decreased compared to that of the control group (Figure 2D).

### 3.3. IDH3α Regulates the EMT in UCC

Next, lentivirus was used to knock down IDH3α in CaSki cells, and differentially expressed genes (DEGs) were analyzed by using RNA-seq (Figure S1A,B). We undertook KEGG pathway and GO analyses that focused on the cell migration and invasion, in response to the immune system (Figure S1C,D). We also found a significant difference in the expression of epithelial–mesenchymal transition (EMT)-related markers (Figure S1E).

**Figure 3:**
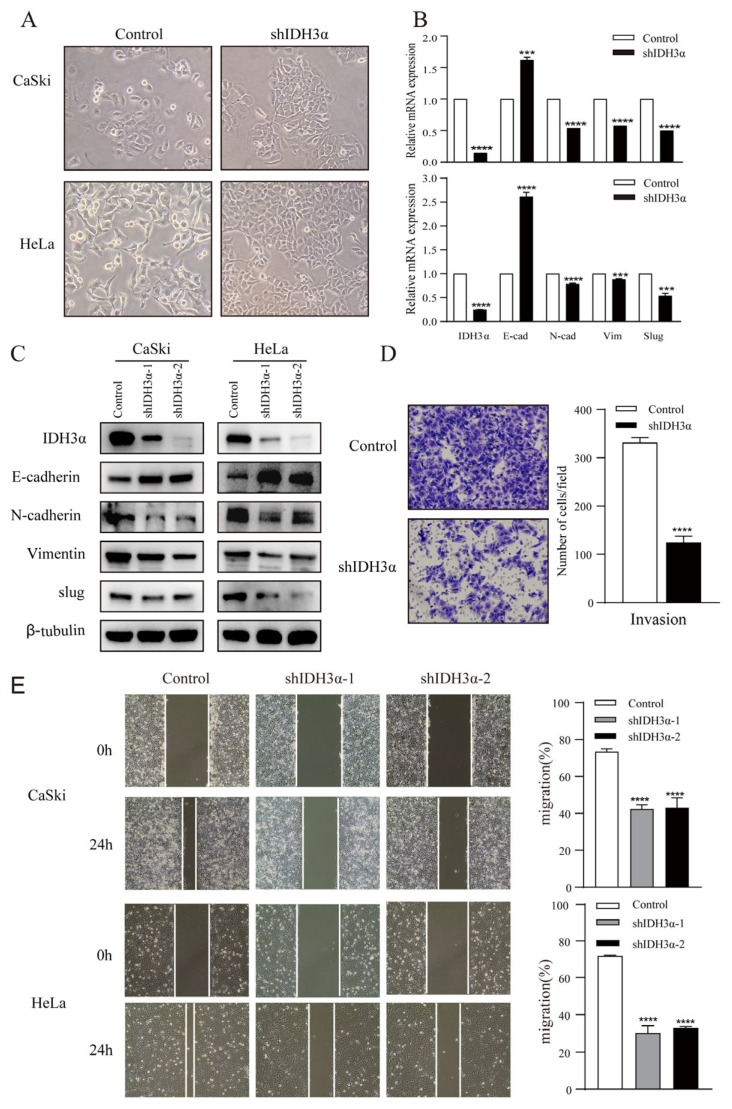

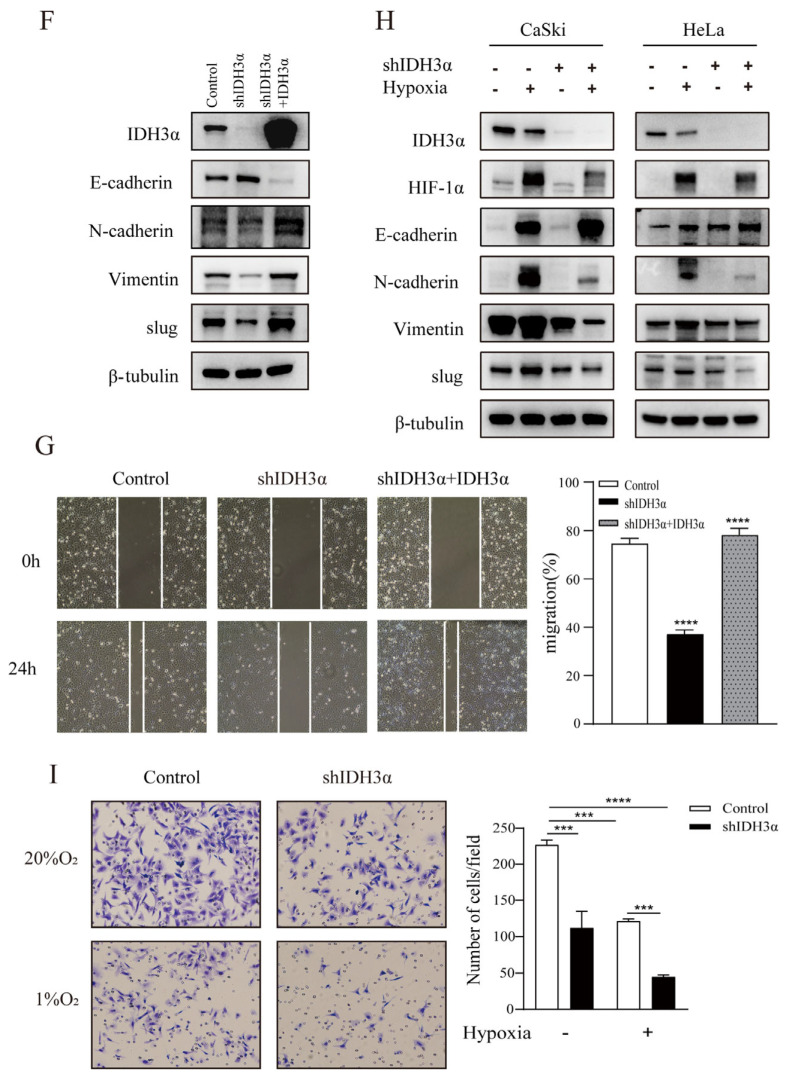
IDH3α knockdown inhibited the EMT and disrupted EMT-related biomarkers in UCC cells. (**A**) Morphological changes in CaSki and HeLa cells were observed under a phase contrast microscope. (**B**,**C**) qPCR (**B**) and WB (**C**) were used to detect E-cadherin, N-cadherin, vimentin, and slug in UCC cells. (**D**) Scratch assay and histogram statistics (right) show that the downregulation of IDH3α inhibited CaSki and HeLa cell migration ability. (**E**) Transwell cell invasion assays and histogram statistics (right) used for HeLa-shIDH3α and control cells. (**F**) WB was used to detect EMT-related marker expression after knocking down IDH3α expression and re-expressing it in CaSki cells. (**G**) The scratch assay was used to detect cell migration ability after knocking down IDH3α expression and re-expressing it in CaSki cells. (**H**) Expression of EMT-related markers under hypoxia was detected by using WB. (**I**) The invasion ability of cervical cancer cells detected by the transwell assay under hypoxia. Data are shown as mean ± SD. *** *p* < 0.001 and **** *p* < 0.0001.

In order to further understand the relationship between IDH3α and the EMT, we observed the morphology of cervical cancer cells. We found that cells were morphologically more likely to adhere tightly together, rather than spread around, in the group with downregulated IDH3α expression (Figure 3A). Using RNA-seq, qPCR, and WB detection, we confirmed that silencing IDH3α upregulated the expression of E-cadherin and downregulated the expression of N-cadherin, vimentin, and slug (Figure 3B,C). Then, we observed that the downregulation of IDH3α in HeLa cells significantly suppressed the invasive ability of cancer cells (Figure 3D). The scratch assay results showed that silencing IDH3α significantly inhibited the migration ability of UCC cells (Figure 3E). In addition, we decreased IDH3α expression and re-expressed it in the same cells, which significantly restored EMT-related marker expression (Figure 3F), and scratch experiments demonstrated the restoration of the migratory ability (Figure 3G). These data suggest that the downregulation of IDH3α inhibits the migration and invasion of UCC cells.

Considering that tumor cells grow under hypoxic conditions, we further examined the regulatory effects of IDH3α on the EMT in this environment. Under hypoxia (1% O_2_), the downregulation of IDH3α could further upregulate the expression of E-cadherin by reducing HIF-1α expression and downregulating the expression of vimentin, N-cadherin, and slug expression in WB. (Figure 3H). Transwell assays showed that the knockdown of IDH3α further inhibited the invasive ability of tumor cells under hypoxia (Figure 3I).

### 3.4. IDH3α Controls Intracellular Redox Status and Glycolysis Ability

**Figure 4:**
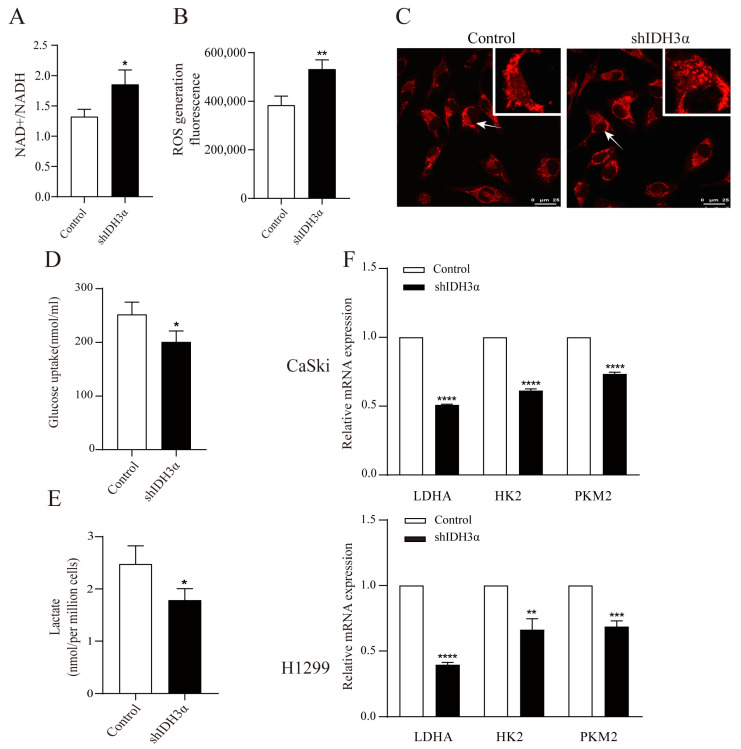
The effects of IDH3α knockdown on redox status and glycolysis ability. (**A**) NADH oxidase activity was assessed in H1299 cells. (**B**) ROS detection was assessed by using the Cellular Reactive Oxygen Species Detection Assay Kit (Red Fluorescence) in H1299 cells. (**C**) Confocal microscopy visualization of mitochondrial morphology after mitochondrial staining with the mitochondrial tracker red CMXRos in HeLa cells. Arrows represent mitochondria. (**D**) Glucose uptake in H1299-shIDH3α and control cells. (**E**) Lactate concentration in H1299-shIDH3α and control cells. (**F**) Glycolysis-related markers were detected by quantitative PCR. Data are shown as mean ± SD. * *p* < 0.05, ** *p* < 0.01, *** *p* < 0.001 and **** *p* < 0.0001.

We detected the redox status in shIDH3α cells by using metabolite kits and found that the knockdown of IDH3α increased the ratio of NAD+/NADH (Figure 4A) and total ROS levels (Figure 4B). In addition, mitochondrial morphology was stained by the Mito Tracker Red CMXRos dye and visualized by confocal microscopy. We found that more mitochondrial fragments were observed in shIDH3α cells, suggesting that mitochondrial fission increased (Figure 4C). These results suggest that the knockdown of IDH3α inhibited mitochondrial function.

We determined whether IDH3α could affect the glycolysis ability by detecting the glucose uptake and lactate production in cancer cells, and it was found that the knockdown of IDH3α reduced cellular glucose uptake (Figure 4D) and lactate production (Figure 4E). Next, we determined the effect of IDH3α on glycolytic enzymes expression. The mRNA expression of glycolytic enzymes (LDHA, HK2, and PKM2) was decreased in UCC and LUAD cells after the inhibition of IDH3α expression (Figure 4F).

### 3.5. Downregulation of IDH3α Expression Increased Sensitivity to Cisplatin

**Figure 5:**
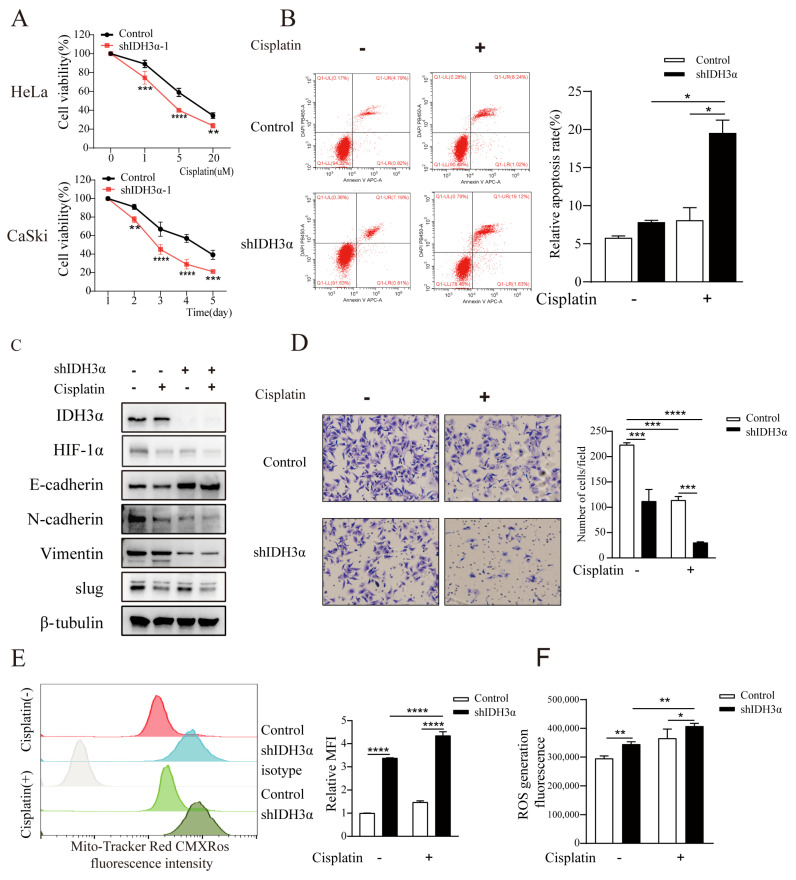
Silencing IDH3α increased sensitivity to cisplatin treatment in cancer cells. (**A**) HeLa cell viability was dose-dependently inhibited after different doses of cisplatin treatment, and CaSki cell viability was time-dependently inhibited after the use of 10 µM cisplatin treatment. (**B**) Cell apoptosis was quantitatively evaluated using annexin V/DAPI flow cytometry after 5 µM cisplatin treatment for 24 h in HeLa cells. (**C**) Western blot analysis of EMT-related markers after 5 µM cisplatin treatment for 24 h in CaSki cells. (**D**) Transwell assay showing that shIDH3α combined with 5 µM cisplatin treatment for 24 h inhibited HeLa cell-invasion ability. € Flow cytometric analysis showed that mitochondrial content (mitochondrial tracker red CMXRos) increased after 10 µM cisplatin treatment for 24 h in CaSki-shIDH3α cells. (**F**) Increased ROS production was detected in HeLa-shIDH3α cells after 10 µM cisplatin treatment for 24 h. * *p* < 0.05, ** *p* < 0.01, *** *p* < 0.001, and **** *p* < 0.0001.

Given the important role of the EMT in chemotherapeutic drug resistance, we assessed the effect of IDH3α knockdown on cisplatin treatment. We found that the downregulation of IDH3α inhibited the viability of UCC cells in a dose- and time-dependent manner using the CCK8 assay (Figure 5A and Figure S2A). Measurements of apoptosis by flow cytometry showed that shIDH3α combined with cisplatin further promoted apoptosis (Figure 5B). The detection of EMT-related markers by WB and transwell experiments showed that shIDH3α combined with cisplatin further inhibited the invasive ability of UCC cells (Figure 5C,D). Studies have shown that cisplatin promotes apoptosis by increasing mitochondrial ROS production [20]. ROS detection found that the mitochondrial content of CaSki cells increased after cisplatin treatment (Figure 5E), and the ROS content was further increased compared to that in the control group (Figure 5F). The results show that silencing IDH3α increased the sensitivity of UCC cells to cisplatin.

### 3.6. IDH3α Knockdown Inhibited UCC Growth In Vivo and Increased CD8^+^ T Cell Infiltration Proportion after Cisplatin Treatment

**Figure 6:**
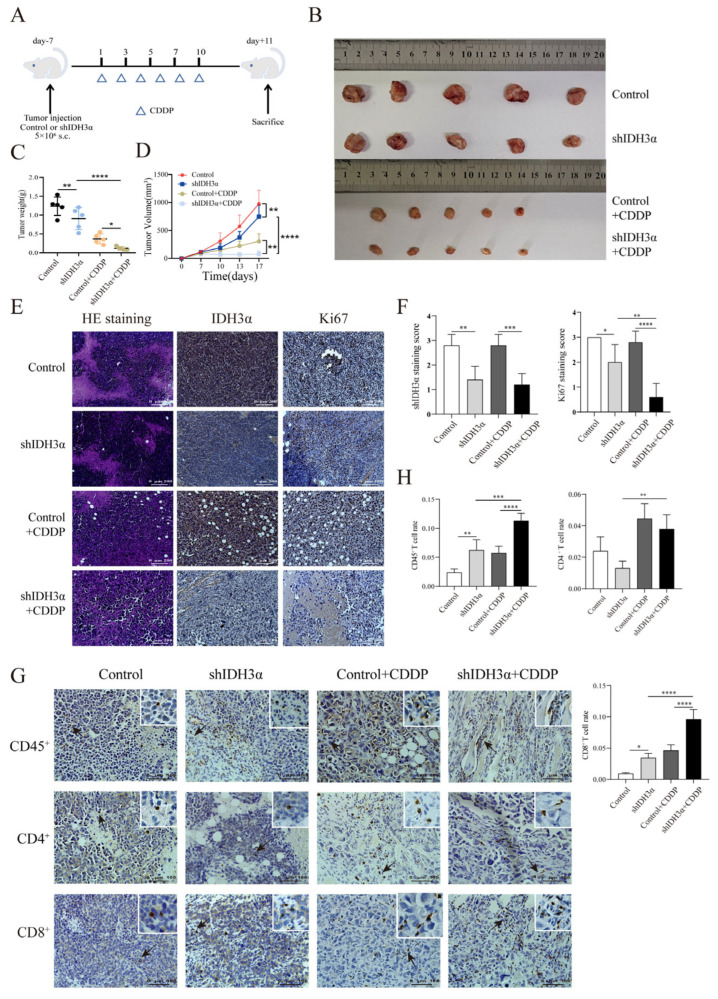
Effects of IDH3α on tumor growth in vivo and CD8^+^T cell infiltration proportion in tumor tissues after cisplatin treatment. (**A**) In vivo experimental design. U14 mouse cervical cancer cells were transduced with lentivirus and injected into the inguinal region of C57BL/6 wild-type mice (5 × 10^6^ per mouse). Cisplatin (5 mg/kg per mouse) was injected intraperitoneally on days 1, 3, 5, 7, and 10. (**B**–**D**) Images of subcutaneous tumor and growth curve of tumor tissue weight and volume (n = 5/group). (**E**,**F**) Tumor tissues were collected, fixed with formaldehyde, sectioned, and examined using H&E staining. Immunohistochemical and quantitative analysis of Ki67 and IDH3α. (**G**,**H**) Immunohistochemical and quantitative analysis of CD45^+^, CD4^+^, and CD8^+^T cells in tumor tissues. * *p* < 0.05, ** *p* < 0.01, *** *p* < 0.001, and **** *p* < 0.0001.

We used lentivirus to transduce mouse UCC U14 cells, verified the transduction effect by Western blotting (Figure S4A), and observed the effect of IDH3α expression on tumor growth after cisplatin treatment. Tumor weights and volumes were significantly reduced in the downregulated IDH3α group compared with those in the control group (Figure 6B–D). Immunohistochemical (IHC) staining of sections and quantitative analysis showed that the expression of the proliferation marker Ki67 was significantly decreased in the shIDH3α and shIDH3α+CCDP groups (Figure 6E,F). One study found that in an orthotopic model of NSCLC, the benefit of cisplatin treatment was associated with increased T lymphocyte infiltration [21]. Our results show that CD45^+^, CD4^+^, and CD8^+^ T cells were increased in tumor tissues after cisplatin treatment, and the proportions of CD45^+^ and CD8^+^ T cells were significantly increased in the shIDH3α+CDDP group (Figure 6G,H). These results suggest that silencing IDH3α may promote cisplatin chemosensitivity by affecting the proportion of CD8^+^ T cells in the immune microenvironment.

### 3.7. IDH3α Knockdown Enhanced Chemoimmunotherapy Sensitivity by Activating cGAS–STING Pathway

**Figure 7:**
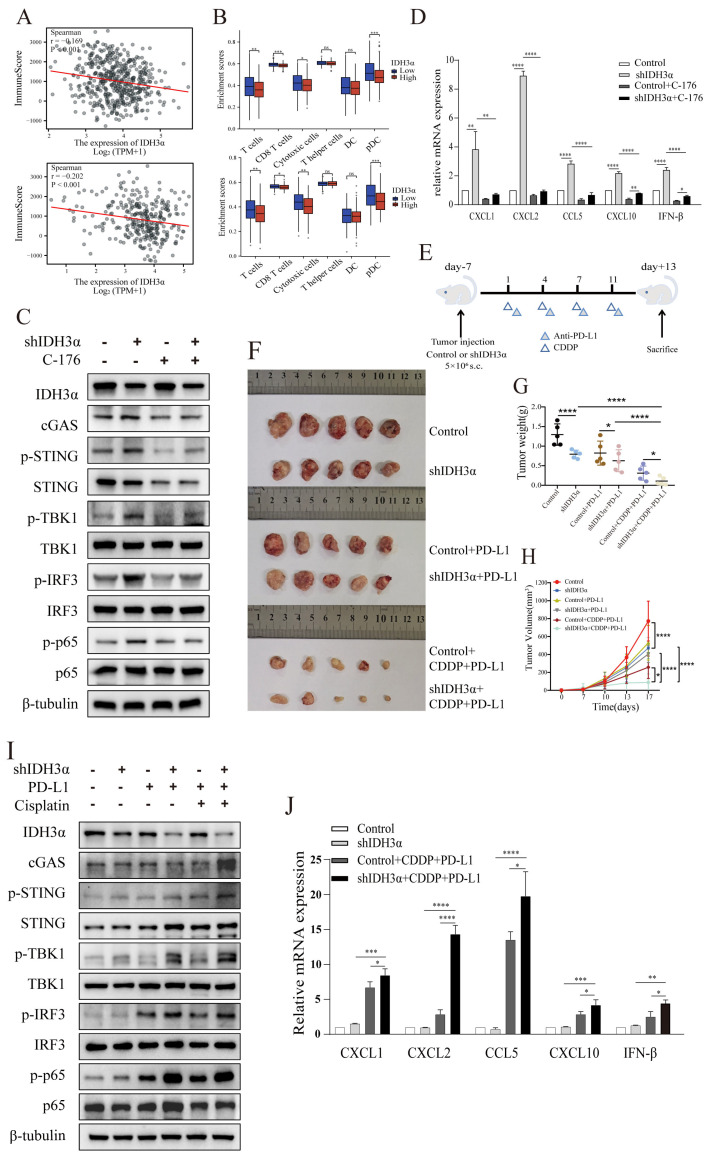

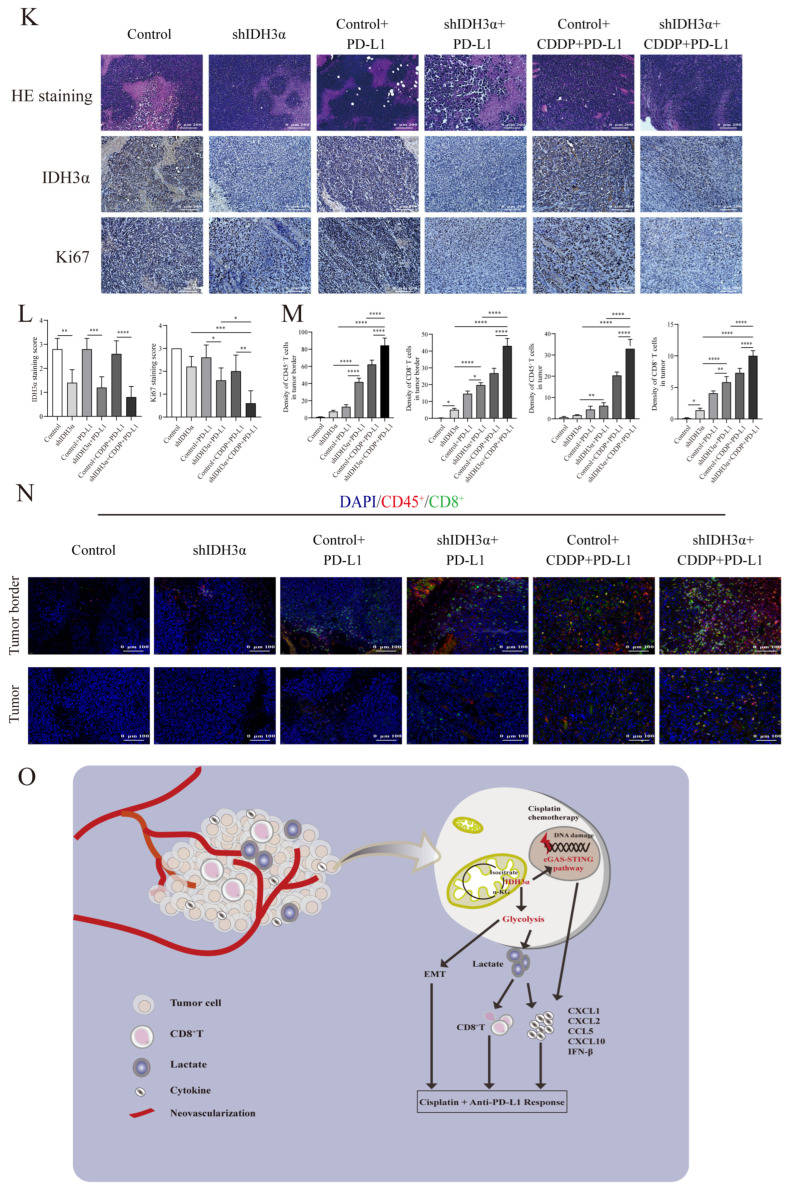
The depletion of IDH3α sensitized tumors to cisplatin + anti-PD-L1 chemoimmunotherapy and activated the cGAS–STING pathway. (**A**) Analysis of correlation between IDH3α and immune score for lung cancer (upper) and UCC (lower). Dots represent patients. (**B**) Analysis of the effect of IDH3α on the infiltration proportion of various immune cells for lung cancer (upper) and UCC (lower). (**C**) The WB detection of cGAS–STING pathway-related protein expression after 20 µM C-176 treatment for 24 h. (**D**) qPCR analysis of mRNA expression levels of CXCL1, CXCL2, CCL5, CXCL10, and IFN-β after 20 µM C-176 treatment for 24 h. (**E**) Experimental design to investigate the role of IDH3α in anti-PD-L1 and/or cisplatin treatment. U14 cells were transfected with lentivirus and injected into the inguinal region of C57BL/6 wild-type mice (5 × 10^6^ cells per mouse). Cisplatin (5 mg/kg per mouse) was injected intraperitoneally on days 1, 4, 7, and 11, and the anti-PD-L1 antibody (100 μg per mouse) was injected intraperitoneally on days 2, 5, 8, and 12. (**F**–**H**) Images of subcutaneous tumor growth in C57BL/6J mice, as well as statistical analysis of tumor tissue weight and volume (n = 5/group). (**I**) WB detection of cGAS–STING-pathway-related protein expression in the tissues of each experimental treatment group. (**J**) qPCR analysis of CXCL1, CXCL2, CCL5, CXCL10, and IFN-β mRNA expression levels in the tissues of each experimental treatment group. (**K**,**L**) H&E, IHC staining and quantitative analysis of Ki67 and IDH3α in each experimental group. (**M**,**N**) Immunofluorescence and quantitative analysis of D45^+^ and CD8^+^ immune cells in the tumors of each treatment group. CD45^+^ (red) and CD8^+^ (green) cells. (**O**) Schematic diagram of the role of IDH3α in regulating tolerance to chemotherapy and immunotherapy. * *p* < 0.05, ** *p* < 0.01, *** *p* < 0.001 and **** *p* < 0.0001.

To further explore the mechanism of IDH3α regulation of the immune microenvironment, we analyzed the correlation of IDH3α with lung cancer and UCC immune scores in the TCGA database using the R language estimate package. The data show that the lower the IDH3α expression, the higher the immune score (Figure 7A). We used the GSVA package to analyze the differential immune cell infiltration between the IDH3α-high and -low expression groups, and the results show that the IDH3α-low expression group had a higher infiltration of T cells, CD8^+^ T cells, cytotoxic T cells, and pDC (Figure 7B). As shown in Figure S1C,D, we found that cytokine–cytokine receptor interaction and type I interferon signaling pathway ranked top in the enrichment analysis. It has been reported that STING activates both IRF3 and NF-κB, which induce type I IFNs and other cytokines, such as CXCL1, CXCL2, CCL5, and CXCL10 [13,22]. Next, we used WB to detect cGAS–STING-pathway-related proteins in UCC cells. The experimental results show that the expression of cGAS, p-STING, p-TBK1, p-IRF3, and p-p65 in the shIDH3α group significantly increased, and the expression of these phosphorylated proteins decreased after STING inhibitor C-176 treatment (Figure 7C and Figure S3A). Then, we detected type I IFNs and other cytokines, which showed that silencing of IDH3α in HeLa cells significantly increased the mRNA expression levels of CXCL1, CXCL2, CCL5, CXCL10, and IFN-β, and the expression of IFN-β and cytokines also decreased after treatment with the STING inhibitor C-176 (Figure 7D).

To further verify the relationship between IDH3α expression and the immune microenvironment cell distribution, a tumor-bearing animal model was established according to the experimental scheme shown in Figure 7E, and the effect of IDH3α expression on tumor growth was observed after different treatments. Compared to that in the control group, the tumor weight was significantly reduced (Figure 7G), and tumor growth curves were suppressed in the shIDH3α group (Figure 7H), especially in the shIDH3α+CDDP+PD-L1 group. Then, we detected cGAS–STING-pathway-related protein expression in the tissues of each experimental treatment group. The results show that the expression of cGAS, p-STING, p-TBK1, p-IRF3, and p-p65 increased mostly in the shIDH3α + CDDP + PD-L1 group (7I). As detected by qPCR, significantly increased mRNA expression levels of CXCL1, CXCL2, CCL5, CXCL10, and IFN-β were detected in tumor tissues isolated from the shIDH3α + CDDP + PD-L1 group compared to the control+CDDP+PD-L1 and shIDH3α groups (Figure 7J). Immunohistochemical (IHC) staining of sections and quantitative analysis showed that the expression of the proliferation marker Ki67 was significantly decreased after IDH3α knockdown (Figure 7K,L). Previous studies have reported that CD8^+^T cells play a major role in the effect of tumor immunotherapy [23]. The silencing of IDH3α alone increased CD8^+^T immune cells at the tumor periphery and at the tumor center, while the combination with CDDP+ anti-PD-L1 Abs resulted in significantly higher proportions than treatment alone or control (Figure 7M,N). The results show that silencing IDH3α could effectively trigger the activation of the cGAS–STING pathway and increase immune cell distribution in the TME.

## 4. Discussion

The metabolic reprogramming of cancer cells is an important feature in tumor initiation and progression, which is still not fully understood [24,25]. Aerobic glycolysis is the main metabolic mode of tumors, but the role of oxidative phosphorylation in tumor metabolism and the role of key enzymes of the tricarboxylic acid cycle in the transition to aerobic glycolysis still need to be explored. The IDH family comprises the rate-limiting enzymes of the TCA cycle, which catalyzes the oxidative decarboxylation of isocitrate to α-KG and exhibits different enzymatic properties under the synergistic effect of different cofactors [19]. In contrast, IDH3α has been shown to affect carcinogenesis [26]. Here, we found that the expression of IDH3α was significantly higher in UCC and LUAD tissues than in normal tissues. Furthermore, high levels of IDH3α are associated with poor prognosis in patients with UCC and LUAD. To understand the effect of IDH3α on the biological phenotype of tumors, we used lentivirus to knock down IDH3α expression and found that the proliferation, apoptosis, migration, and invasion abilities of cancer cells were affected. Therefore, both previous studies and our findings suggest that IDH3α is highly expressed and promotes tumor formation and proliferation [11].

The dysregulation of glycolytic enzymes, such as LDHA [27], HK2 [28], and PKM2 [29], is common in tumors, and their role in the EMT has been extensively reported. There is evidence that increased glucose uptake and enhanced aerobic glycolysis induce intrinsic or acquired resistance to cisplatin in several cancer cell types [30]. In different tumor cells, the regulation of PKM2 expression reduces glucose uptake and lactate and ATP production, thereby increasing the sensitivity of cells to cisplatin [31,32]. The inhibition of the glycolytic pathway increases the sensitivity of drug-resistant ovarian cancer cells to cisplatin [33,34]. Many studies have shown that the EMT can mediate cisplatin resistance through multiple mechanisms that are associated with tumor metastasis and poor prognosis [35,36]. In high-grade serous ovarian cancer (HGSC), sensitivity to cisplatin therapy can be improved by increasing the mitochondrial content or mitochondrial ROS production [20,37]. We have mentioned that IDH3α could promote the EMT by regulating the expression of glycolytic enzymes, and we found that IDH3α knockdown inhibited the resistance of tumor cells to cisplatin by inhibiting glycolysis-related enzymes and inducing ROS production. Therefore, the IDH3α-mediated regulation of chemotherapy’s efficacy is related to the EMT and glycolysis.

The activation of the cGAS–STING pathway in cancer cells combines innate and adaptive immunity, regulates the tumor microenvironment, and can transform cold tumors into hot tumors, increasing the tumor’s sensitivity to immunotherapy [38,39]. It has been reported that STING activates both IRF3 and NF-κB, which mediate immune defense against tumors and viral infections [13]. It can also induce type I IFNs and other cytokines, such as CXCL1, CXCL2, CCL5, and CXCL10 [22]. CXCL1/2 proteins have been found to be potent neutrophil attractors and activators [40], while the increase in CXCL5 and CCL10 could induce the activation and function of cytotoxic T lymphocytes [41,42]. The neutrophil-related chemokines CXCL1 and CXCL2 can be regulated by NF-κB [43]. STING activation enhanced neutrophil migration into the tumor in an NF-κB/CXCL1/2-dependent manner [44]. Moreover, this paper reported that the accumulation of neutrophils in the type I IFN-inflamed tumor microenvironment is essential for the anti-tumor effect mediated by STING-targeting immunotherapy [44]. A study has reported that the increased therapeutic effect of cisplatin was related to the increase in T lymphocyte infiltration [21]. In addition, 5-FU could also promote the anti-tumor effect by activating the STING pathway in the tumor, which could lead to an increase in T cells in the tumor and significantly reduce the depletion of T cells [16]. These studies indicate that the inhibition of IDH3α could promote the activation of the cGAS–STING pathway and increase immune cell infiltration near the tumor tissue, improving the efficacy of immunotherapy.

In addition to the well-known ability of lactate to regulate anti-tumor immune responses [8], increasing evidence indicates that D-2-HG and α-KG can directly affect the number, differentiation, and activation of immune cells in the tumor microenvironment [45,46], whereas α-KG can epigenetically regulate PD-L1 expression [47]. Previous studies have reported that silencing IDH3α could increase α-KG in HeLa cells [11]. Our experimental results showed that IDH3α could regulate the expression of PD-L1 in mouse UCC cells (Figure S4B), which may be one of the reasons why IDH3α could regulate immunotherapy. Characteristics of the tumor microenvironment, including acidity, hypoxia, and increased lactate and reduced glucose concentrations, as well as the acidic extracellular microenvironment, promote cancer development and progression [48]. Therefore, the effects of IDH3α on chemoimmunotherapy are associated with a combination of factors, such as glycolysis and activation of the cGAS–STING pathway, which could be said to result from IDH3α regulation of the acidic TME.

## 5. Conclusions

Overall, high expression of IDH3α promoted metabolic reprogramming and lactate production, which leads to acidic tumor microenvironment disorders. Moreover, the overexpression of IDH3α inhibited cGAS–STING pathway activation, thereby reducing the sensitivity to chemoimmunotherapy (Figure 7O). These results suggest that patients with aberrant expression of IDH3α could be flagged as potentially having tumors that are resistant to neoadjuvant, adjuvant, and salvage chemotherapy and immunotherapy, and such high expression could help to predict prognosis.

## Data Availability

The datasets used and/or analyzed during the current study are available from the corresponding author upon reasonable request.

## Supplementary Materials

The following supporting information can be downloaded at https://www.mdpi.com/article/10.3390/cancers15061802/s1. Figure S1: RNA-seq results; Figure S2: SiHa cells’ viability using CCK8 assay; Figure S3: WB detection of cGAS-STING pathway-related protein expression; Figure S4: IDH3α could regulate the expression level of PD-L1; Figure S5: The uncropped Western blots; Table S1: List of expression primers used in this study.

## Abbreviations

TME: tumor microenvironment; CDDP, cisplatin; ICIs, immune checkpoint inhibitor; PD-1, programmed cell death 1; PD-L1, programmed cell death ligand 1; NSCLC, non-small cell lung cancer; UCC, uterine cervical cancer; cGAS, cyclic GMP-AMP synthase; STING, stimulator of IFN genes; IFN, interferon; cGAMP, circular GMP-AMP; IDH, isocitrate dehydrogenase; α-KG, α-ketoglutarate; UALCAN, University of Alabama at the Birmingham Cancer data analysis portal; KM, Kaplan–Meier; DEGs, differentially expressed genes; RNA-seq, RNA sequencing; GO, gene ontology; BPs, biological processes; MFs, molecular functions; CCs, cellular components; LUAD, lung adenocarcinoma; MOI, multiplicity of infection; qPCR, quantitative polymerase chain reaction; WB, Western blotting; EdU, ethynyl-2’-deoxyuridine; IHC, immunohistochemical; HE, hematoxylin and eosin; D-2-HG, D-2-hydroxyglutarate; TSA, tyramide signal amplification; EMT, epithelial–mesenchymal transition; HIF-1α, hypoxia-inducible factor-1α; TCA, tricarboxylic acid; ROS, reactive oxygen species; CXCL, chemokine (C-X-C motif) ligand; CCL5, chemokine (C-C motif) ligand 5; TBK1, TANK binding kinase 1; IRF-3, interferon regulatory factor 3.
