# Supplementary material for: Inhibition of IDH3α Enhanced the Efficacy of Chemoimmunotherapy by Regulating Acidic Tumor Microenvironments
Source: Cancers (Basel). 2023 Mar 16;15(6):1802. doi: 10.3390/cancers15061802 (PMC10046804; doi:10.3390/cancers15061802)
Supplement: Supplementary file 1 [file cancers-15-01802-s001.zip › cancers-2213350-supplementary.pdf]

Figure S1

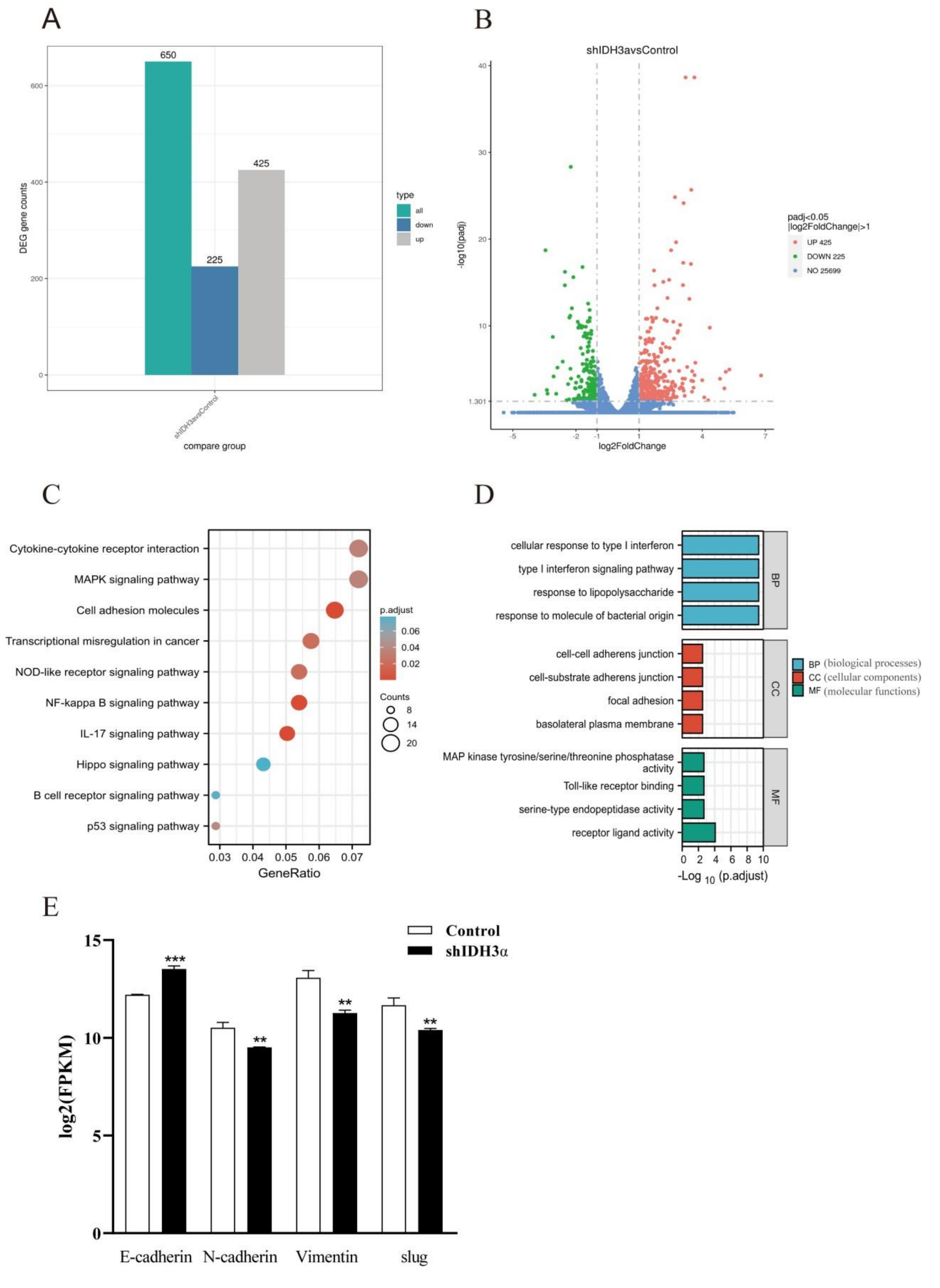

Figure S1. RNA-seq results.

A, Statistical histogram of the number of differential genes (both up- and down-regulated) for each comparative combination (p adj<=0.05, |log2FoldChange|>=1.0.). **B,** Volcano plot of differential gene distribution in treatment and control groups. **C-D,** KEGG**(C)**, and GO **(D)** enrichment analyses were conducted using the R “clusterProfiler” package. BP, biological processes; MF, molecular functions; CC, cellular components. **E,** The expression statistics of EMT markers in the RNA-seq data.

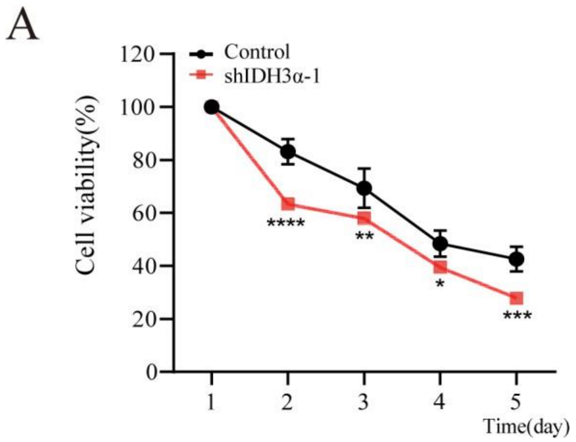

Figure S2. SiHa cells’ viability using CCK8 assay.
SiHa cells’ viability was time-dependently inhibited after the use of 5μM cisplatin treatment.

Figure S3

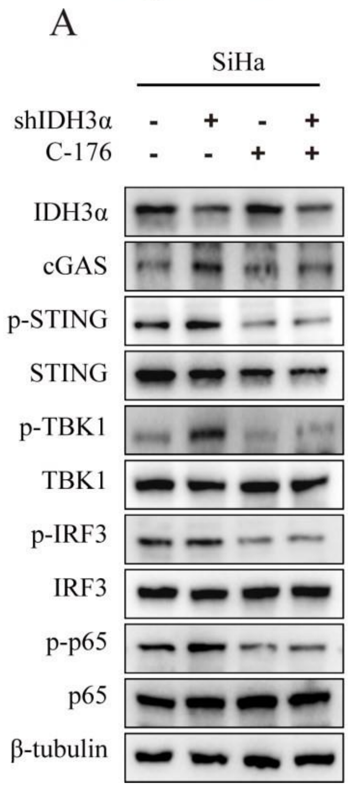

Figure S3. WB detection of cGAS-STING pathway-related protein expression.
A. WB detection of cGAS-STING pathway-related protein expression after 20μM C-176 treatment for 24h in SiHa cells.

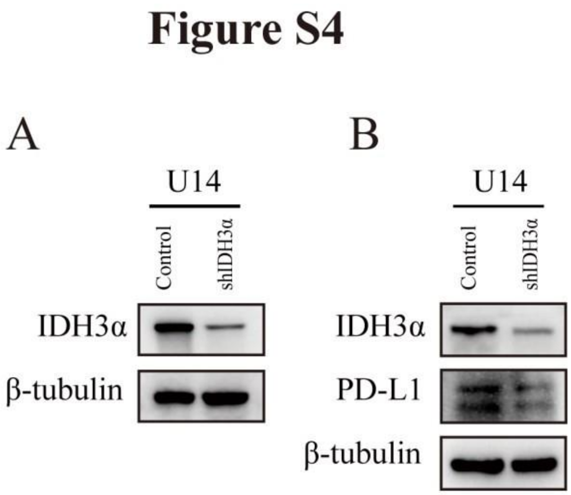

Figure S4. IDH3α could regulate the expression level of PD-L1
A, Validation of IDH3α knockdown mouse cervical cancer stable cell line by WB. **B,** Western blot analysis of the effect of IDH3α-deleted on PD-L1 expression in mouse UCC cells.

Original blots corresponding to Figure 2A:

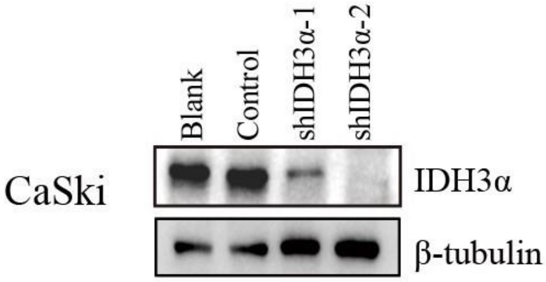

Fig 2A. CaSki IDH3A

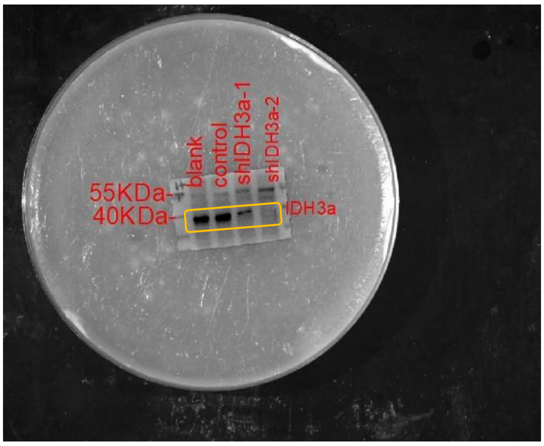

Fig 2A. CaSki β-tubulin

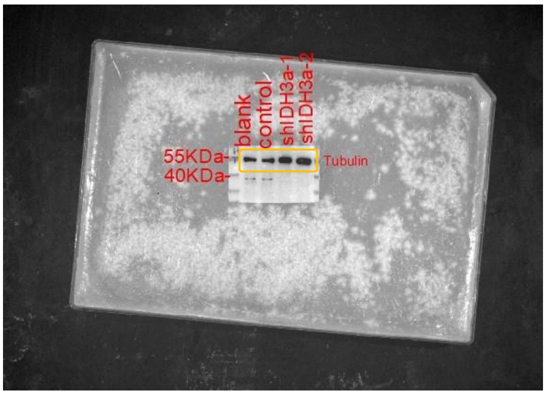

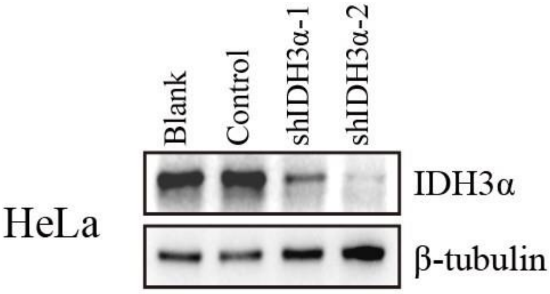

Fig 2A. HeLa IDH3A

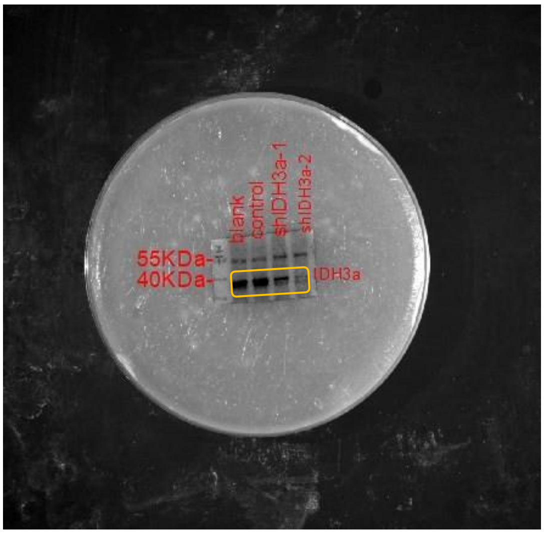

Fig 2A. HeLa β-tubulin

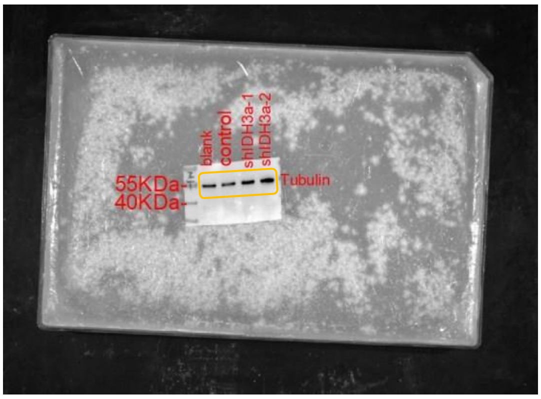

Original blots corresponding to Figure 3C:

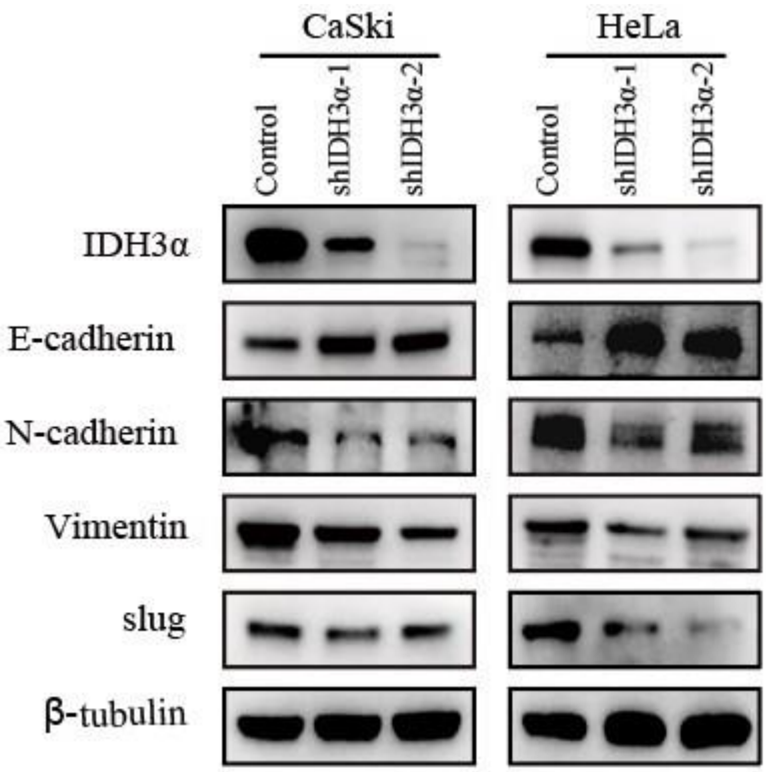

Fig 3C. Caski IDH3A

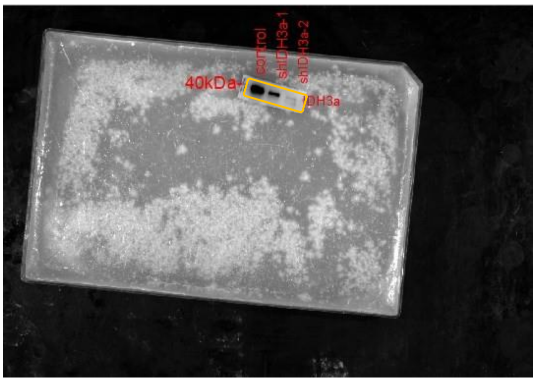

Fig 3C. Caski E-cadherin

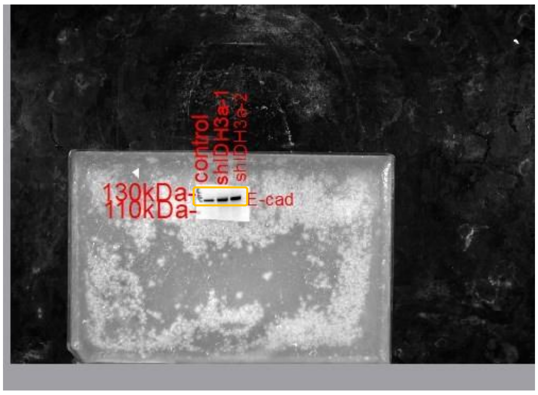

Fig 3C. Caski N-cadherin

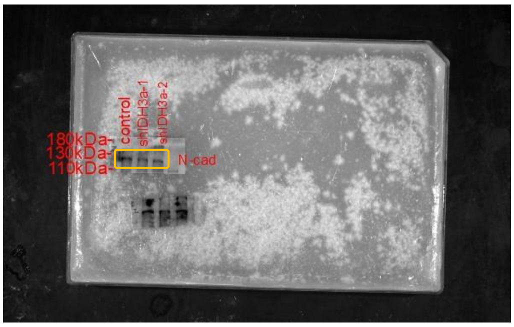

Fig 3C. Caski Vimentin

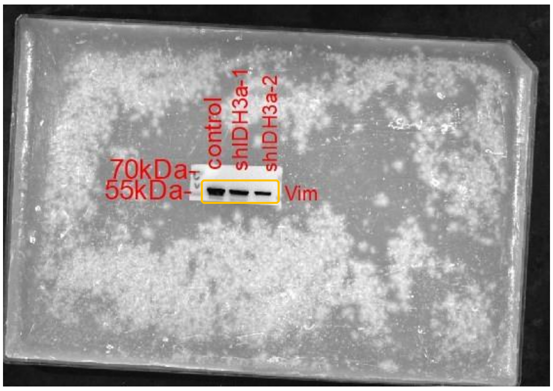

Fig 3C. Caski slug

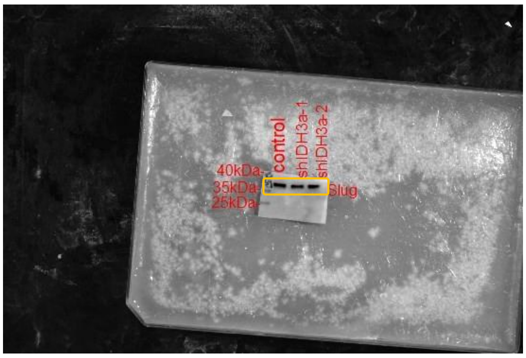

Fig 3C. Caski β-tubulin

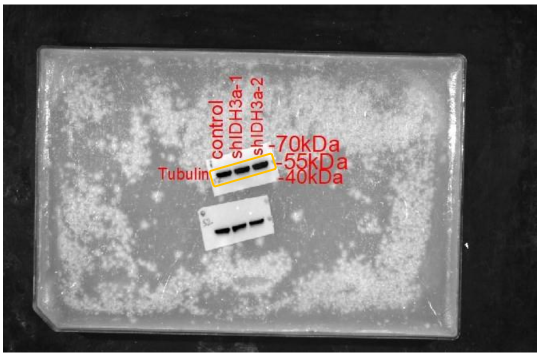

Fig 3C. Hela IDH3A

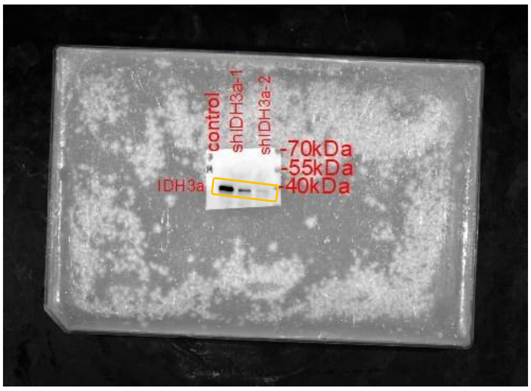

Fig 3C. Hela E-cadherin

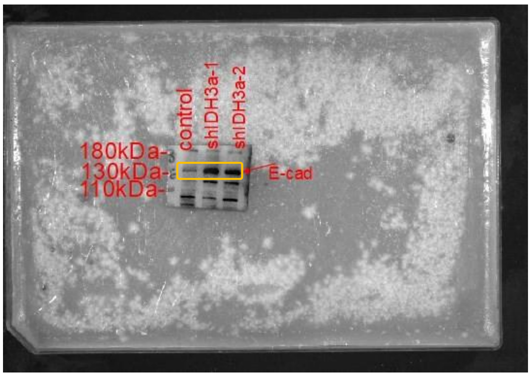

Fig 3C. Hela N-cadherin

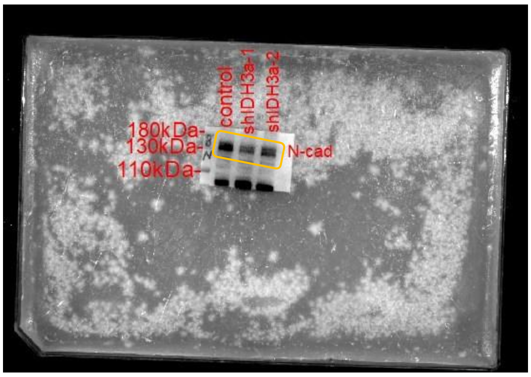

Fig 3C. Hela Vimentin

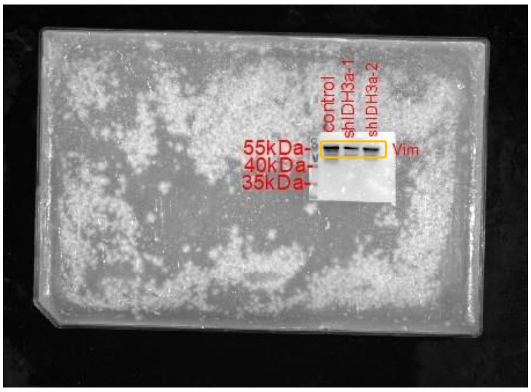

Fig 3C. Hela slug

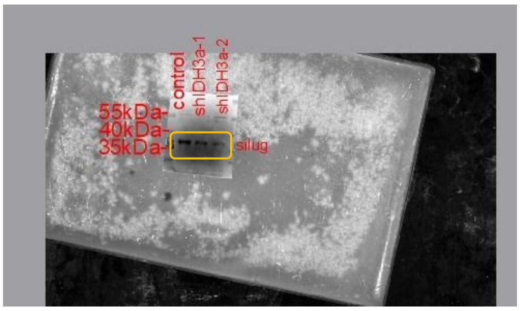

Fig 3C. Hela β-tubulin

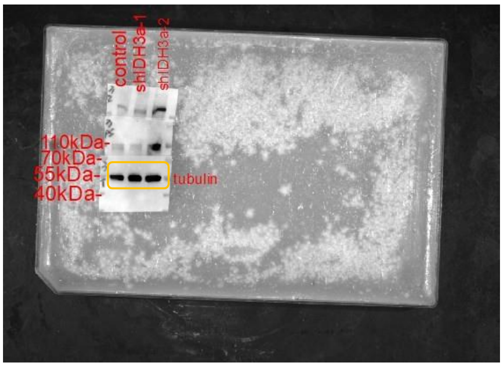

Original blots corresponding to Figure 3F:

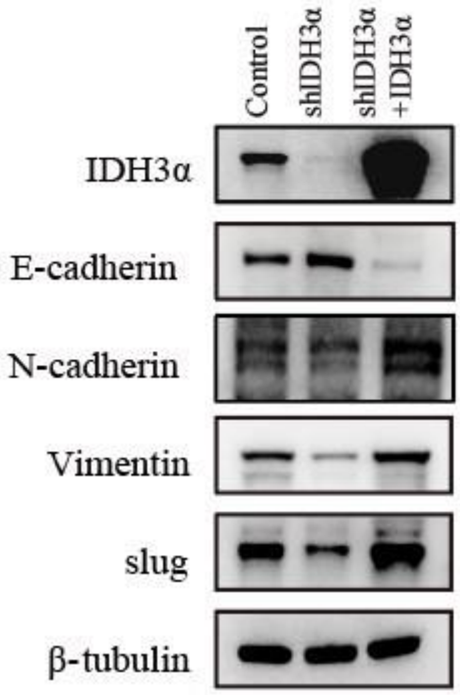

Fig 3F. Caski IDH3A

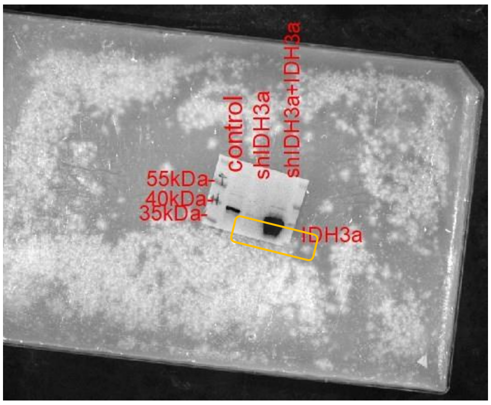

Fig 3F. Caski E-cadherin

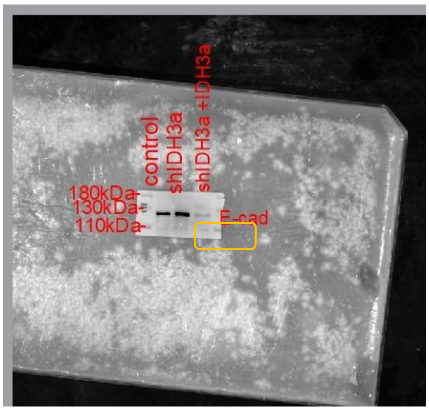

Fig 3F. Caski N-cadherin

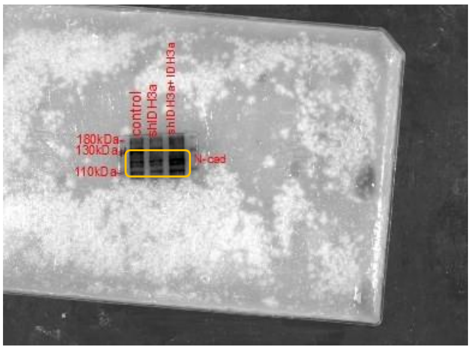

Fig 3F. Caski Vimentin

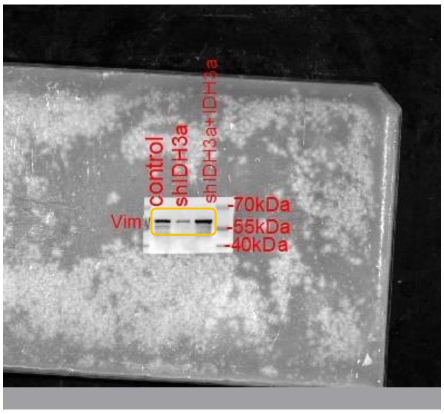

Fig 3F. Caski slug

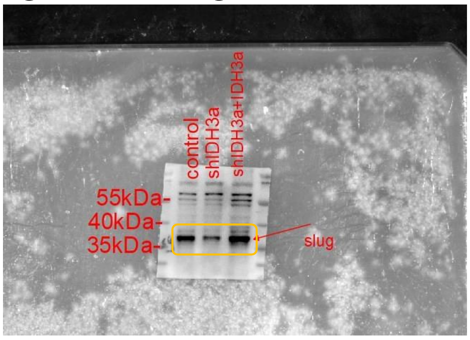

Fig 3F. Caski β-tubulin

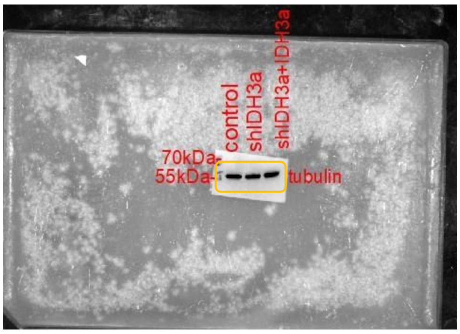

Original blots corresponding to Figure 3H:

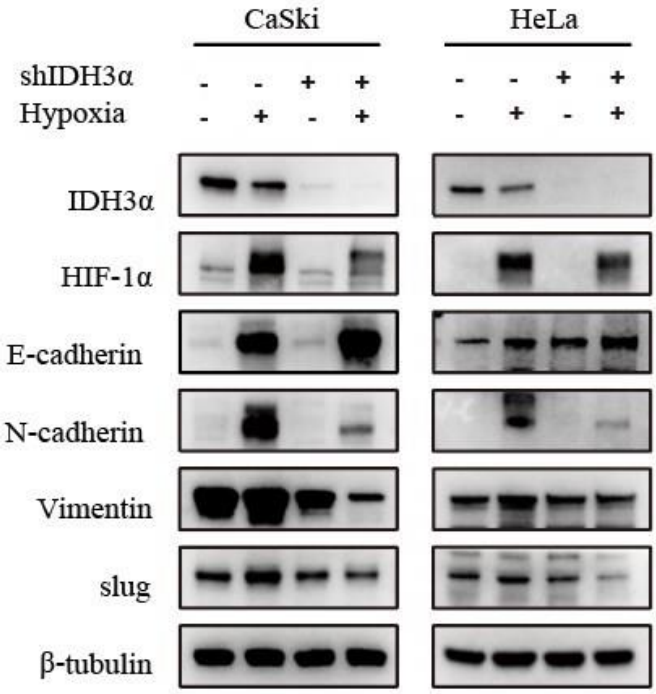

Fig 3H. Caski IDH3A

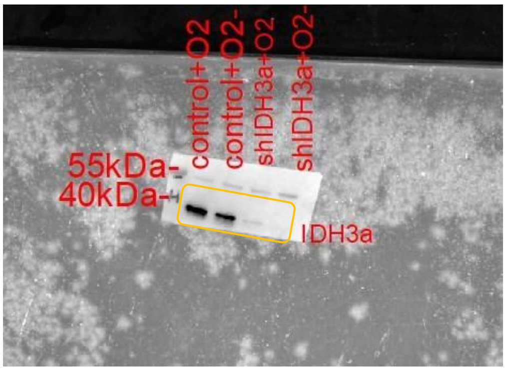

Fig 3H. Caski HIF-1α

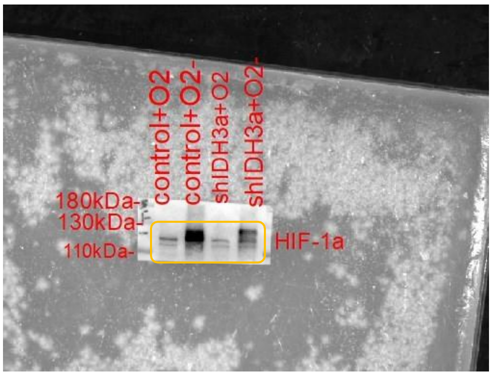

Fig 3H. Caski E-cadherin

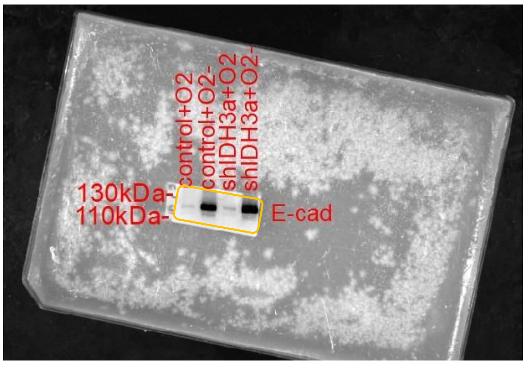

Fig 3H. Caski N-cadherin

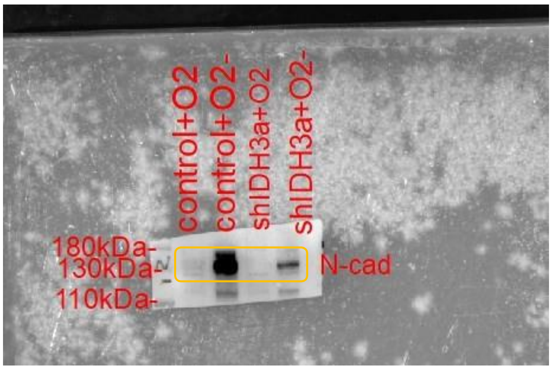

Fig 3H. Caski Vimentin

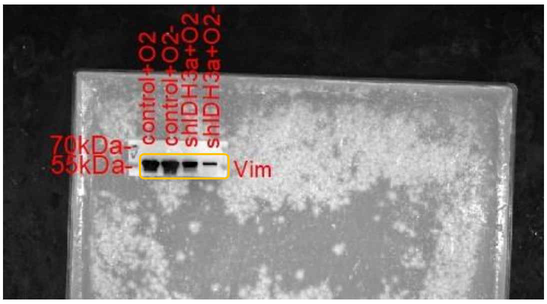

Fig 3H. Caski slug

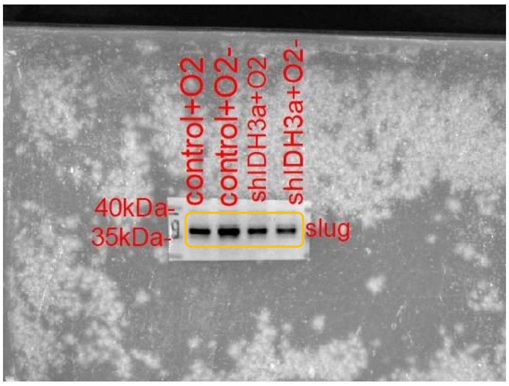

Fig 3H. Caski β-tubulin

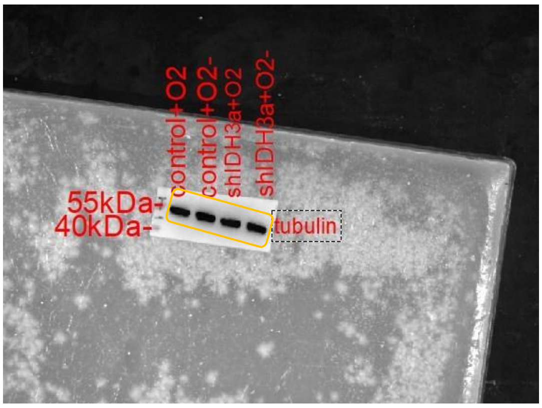

Fig 3H. Hela IDH3A+β-tubulin

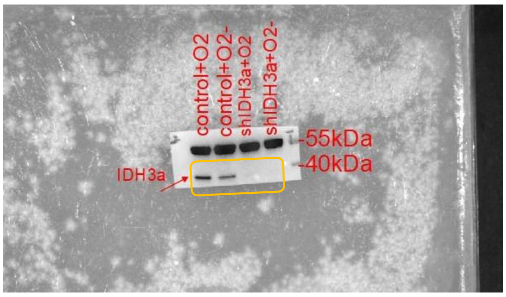

Fig 3H. Hela HIF-1α

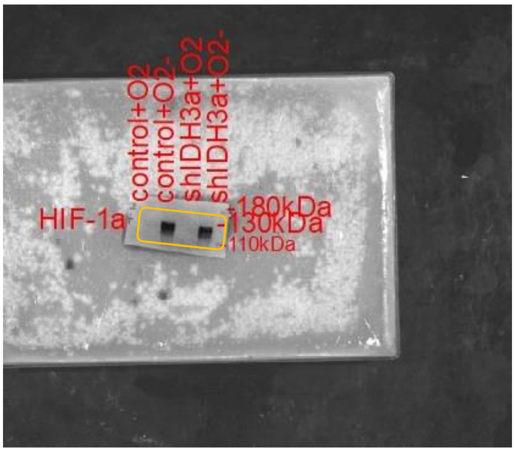

Fig 3H. Hela E-cadherin

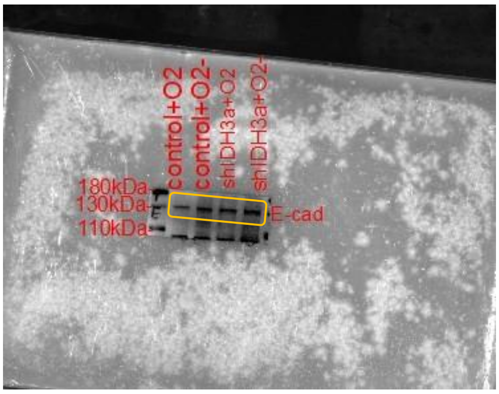

Fig 3H. Hela N-cadherin

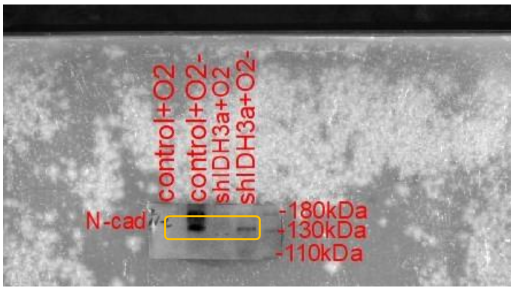

Fig 3H. Hela Vimentin

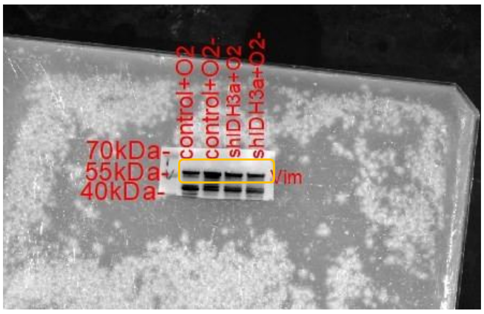

Fig 3H. Hela slug

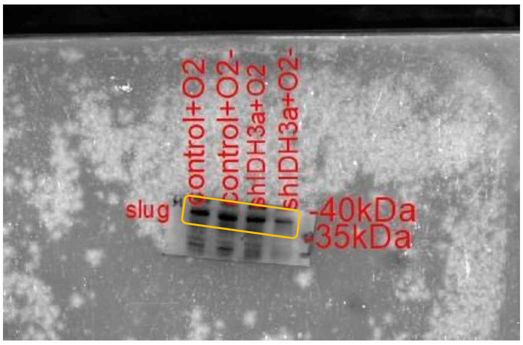

Fig 3H. Hela β-tubulin

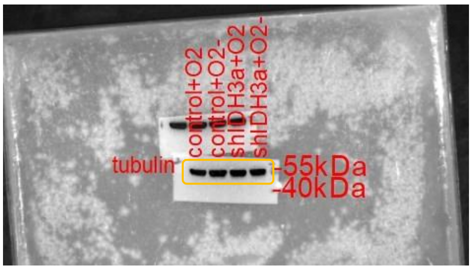

Original blots corresponding to Figure 4D:

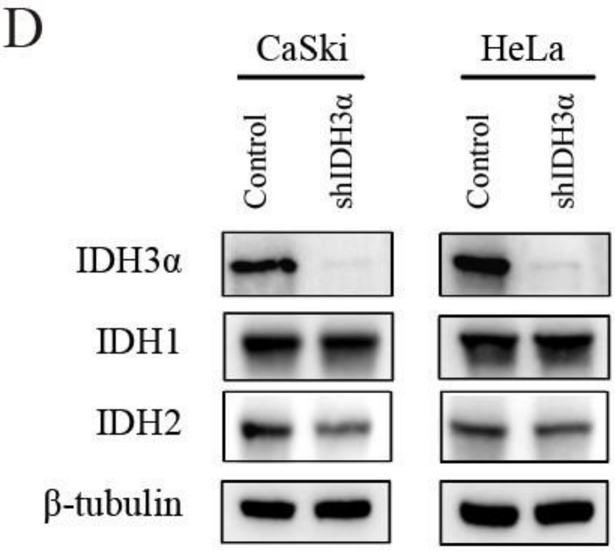

Fig 4D. Caski IDH3A

Fig 4D. Caski IDH1

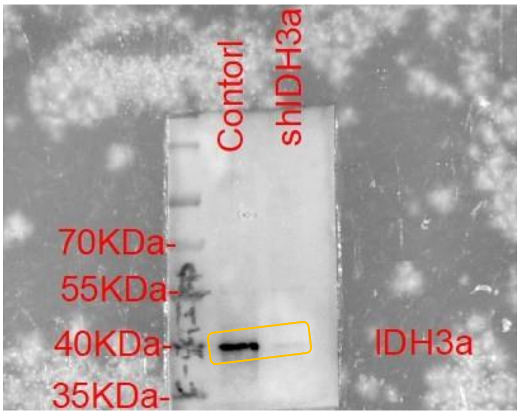

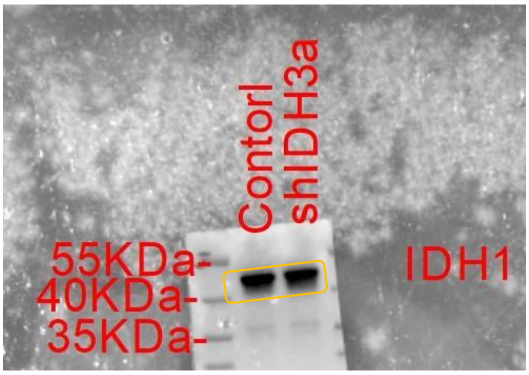

Fig 4D. Caski IDH2

Fig 4D. Caski tubulin

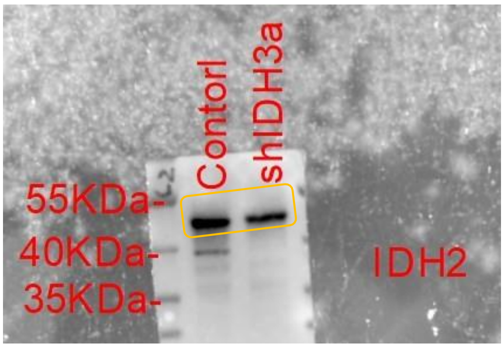

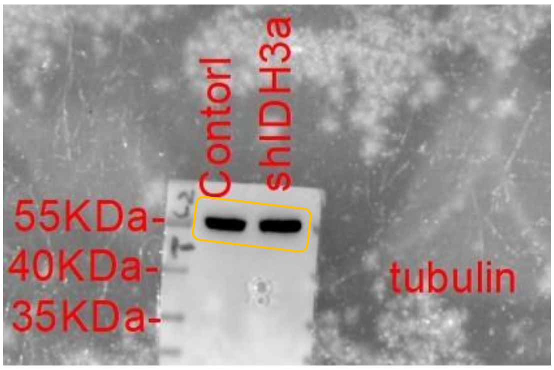

Fig 4D. HeLa IDH3A

Fig 4D. HeLa IDH1

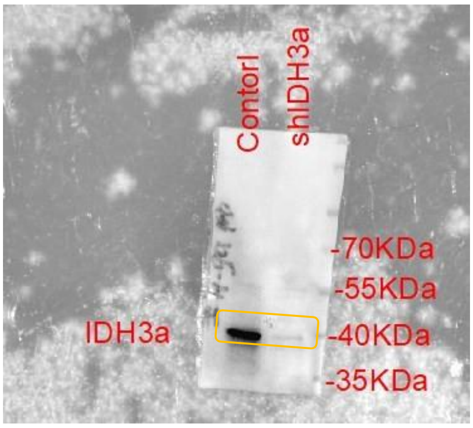

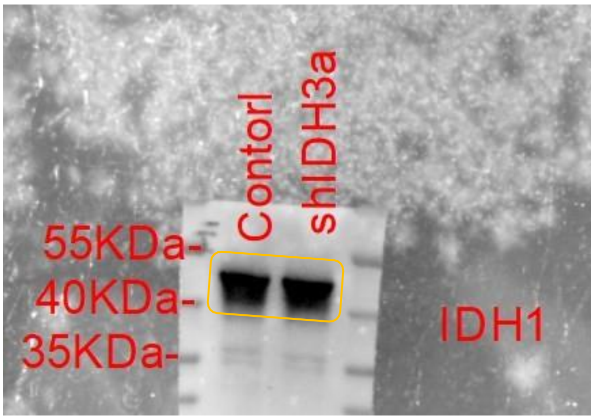

Fig 4D. HeLa IDH2

Fig 4D. HeLa tubulin

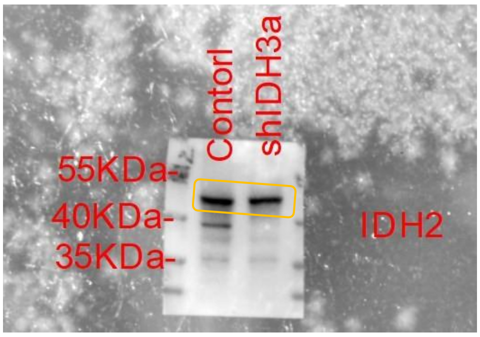

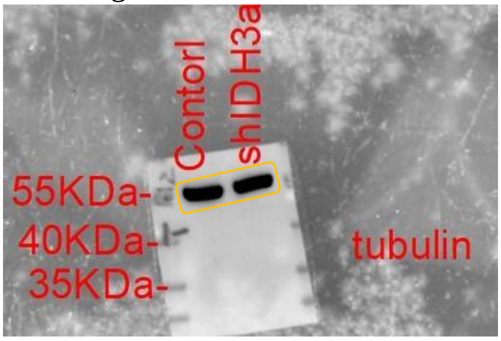

Original blots corresponding to Figure 5C:

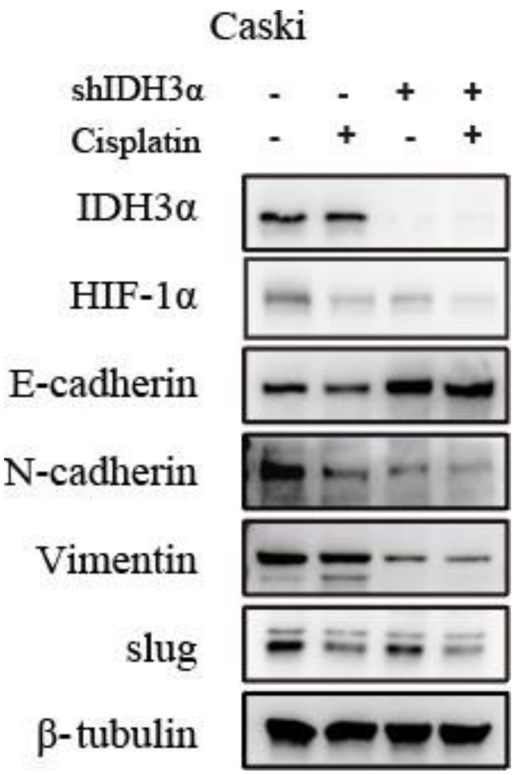

Fig 5C. Caski IDH3A

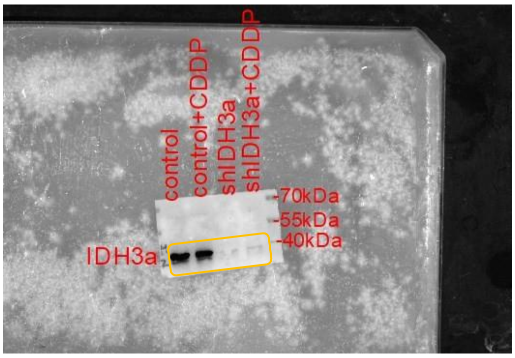

Fig 5C. Caski HIF-1α

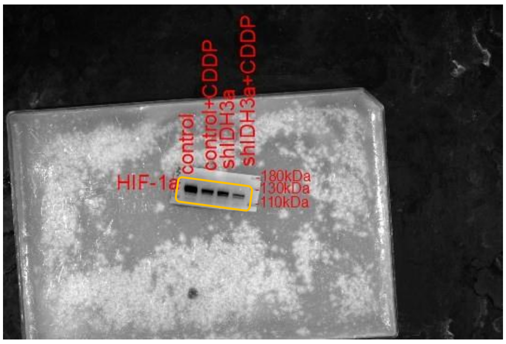

Fig 5C. Caski E-cadherin

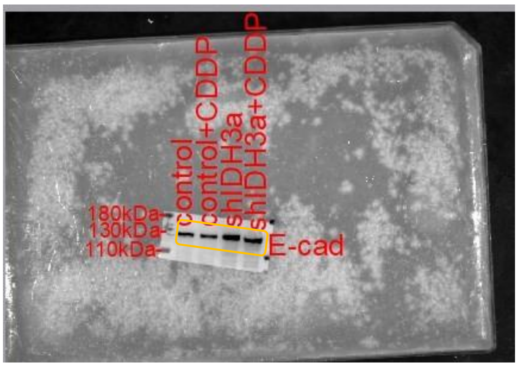

Fig 5C. Caski N-cadherin

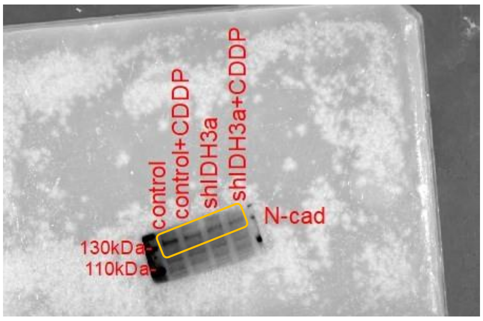

Fig 5C. Caski Vimentin

Fig 5C. Caski slug

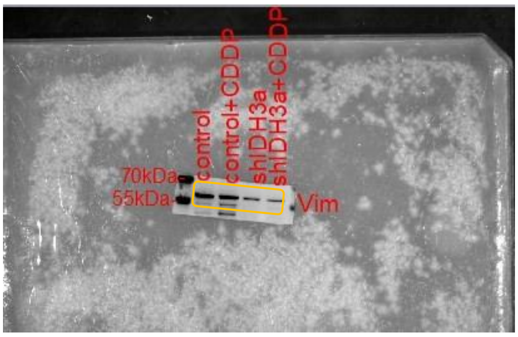

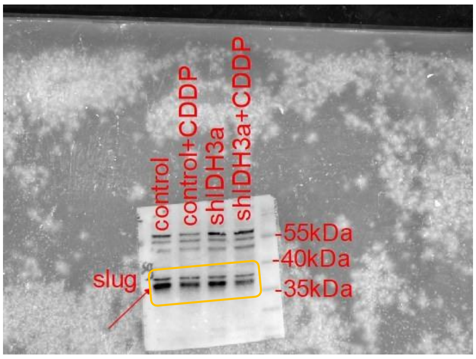

Fig 5C. Caski β-tubulin

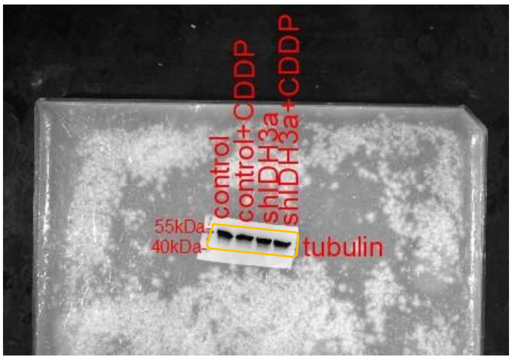

Original blots corresponding to Figure 7C:

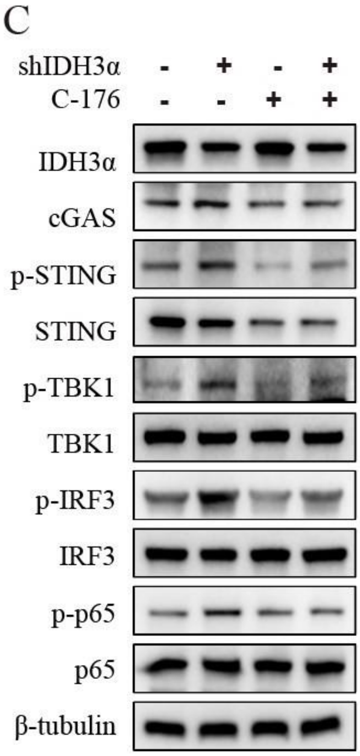

Fig 7C. HeLa IDH3A

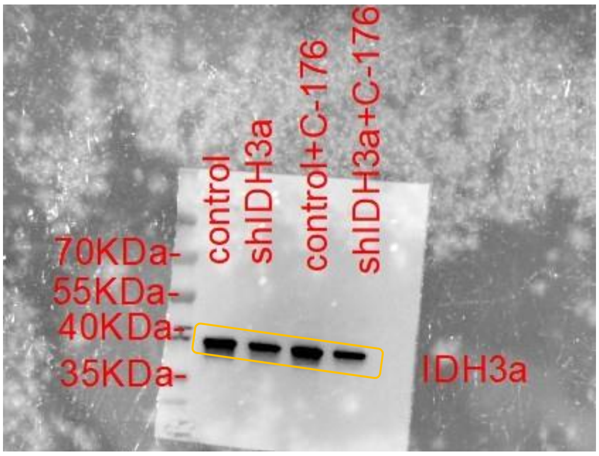

Fig 7C. HeLa cGAS

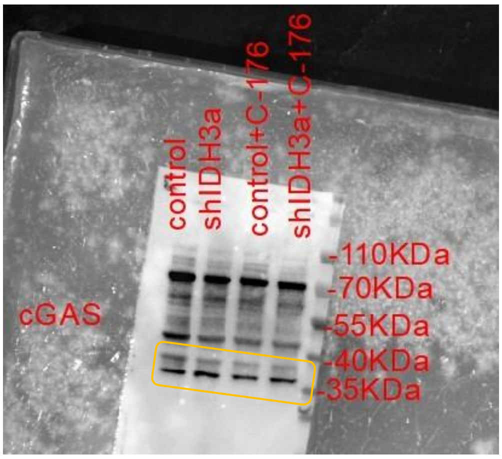

Fig 7C. HeLa p-STING

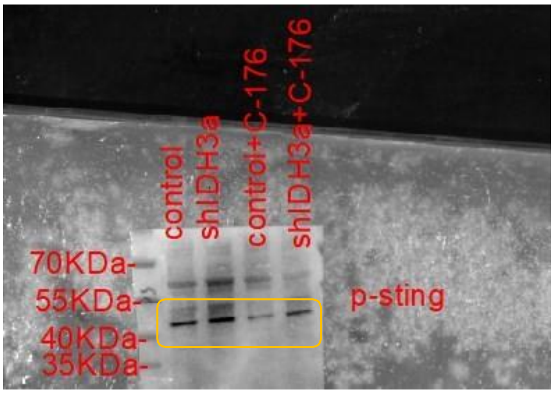

Fig 7C. HeLa STING

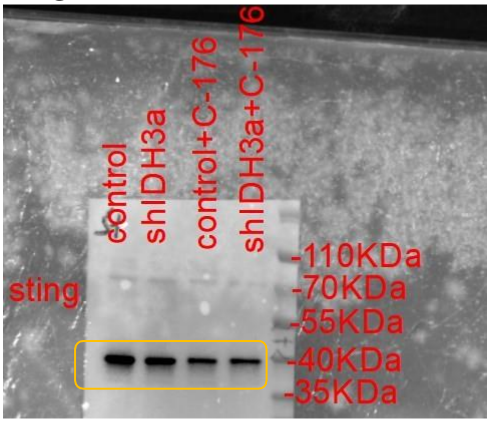

Fig 7C. HeLa p-TBK1

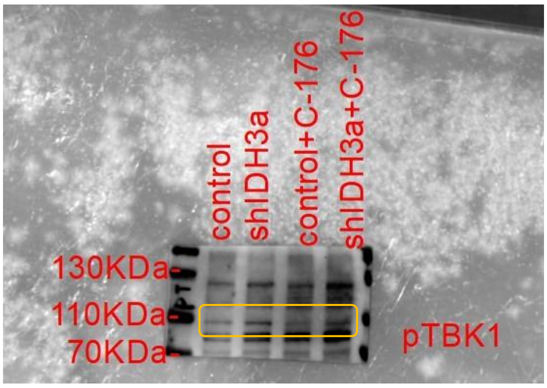

Fig 7C. HeLa TBK1

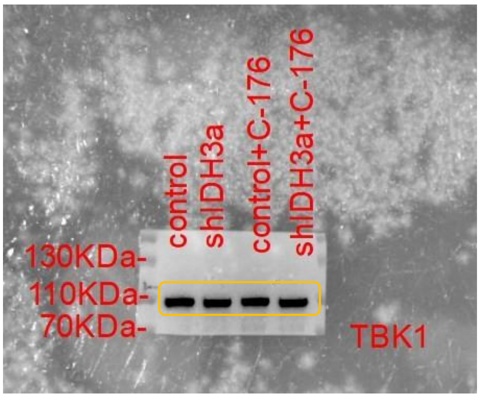

Fig 7C. HeLa p-IRF3

Fig 7C. HeLa IRF3

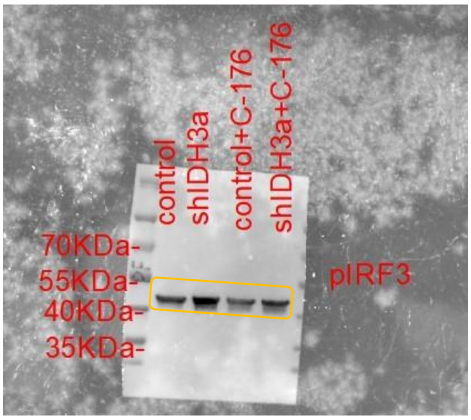

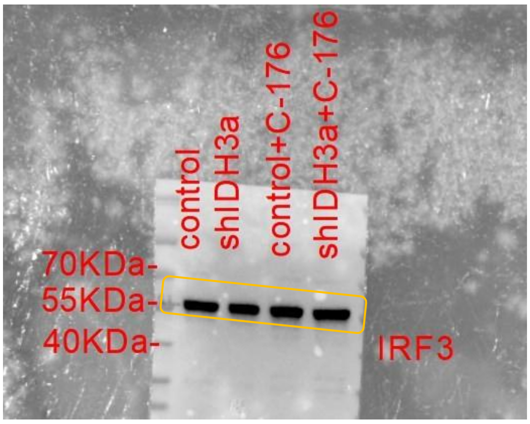

Fig 7C. HeLa p-p65

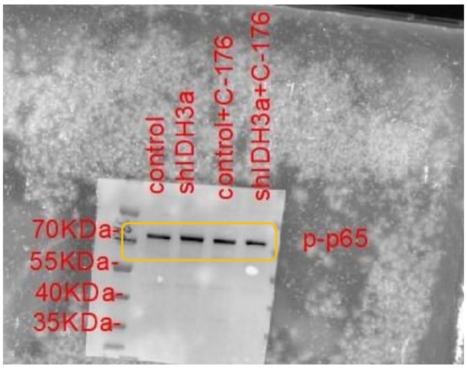

Fig 7C. HeLa p65

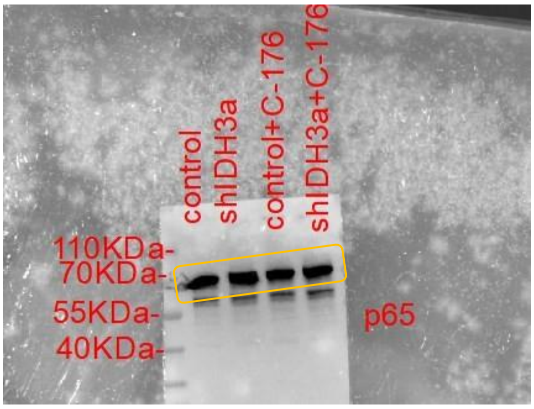

Fig 7C. HeLa β-tubulin

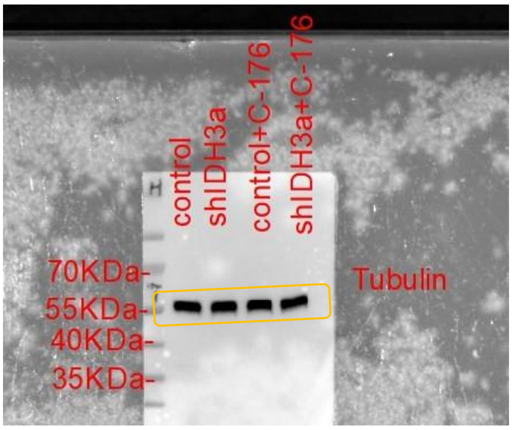

Original blots corresponding to Figure 7I:

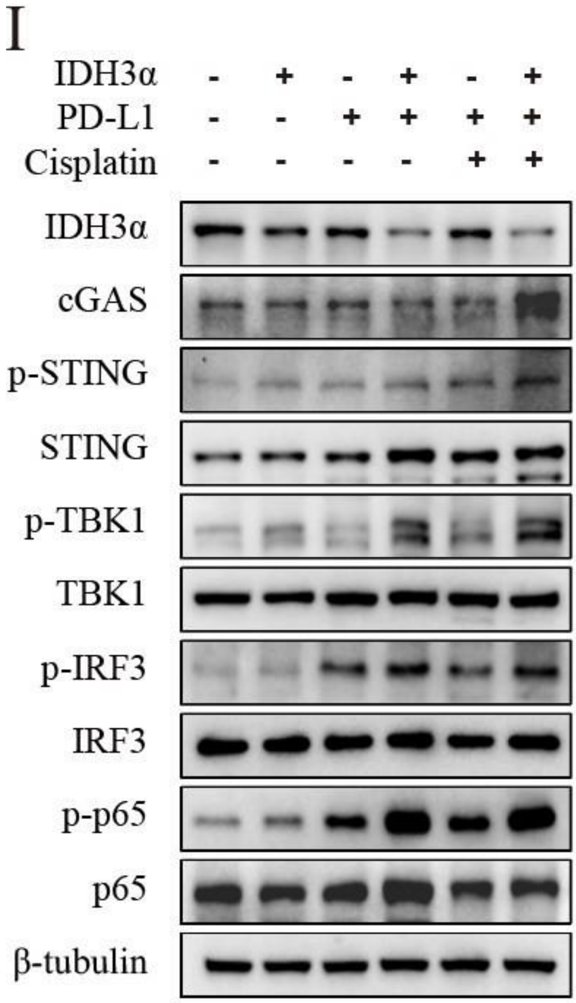

Fig 7I. IDH3A

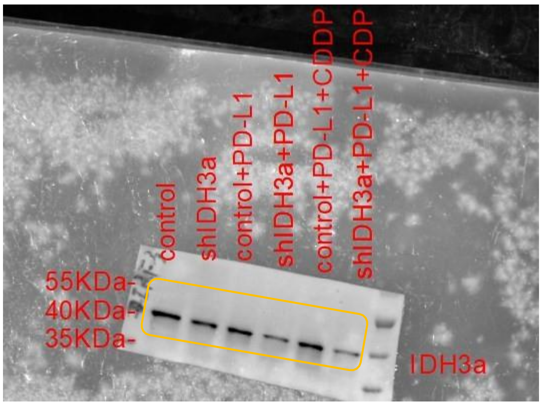

Fig 7I. cGA

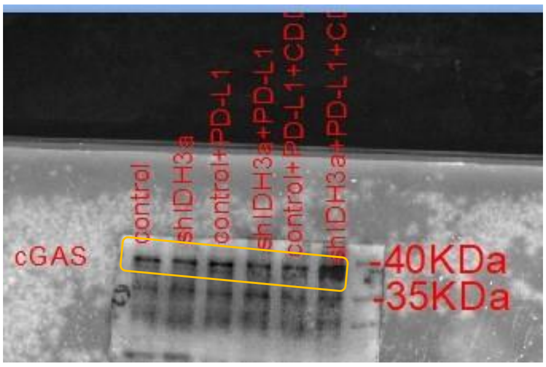

Fig 7I. p-STING

Fig 7I. STING

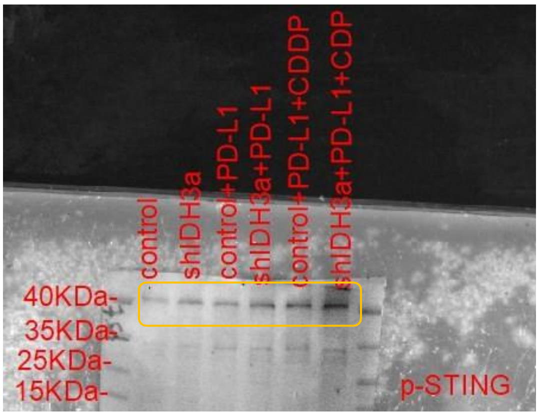

Fig 7I. p-TBK1

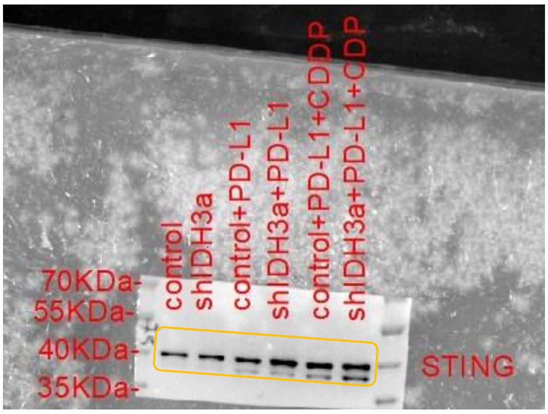

Fig 7I. TBK1

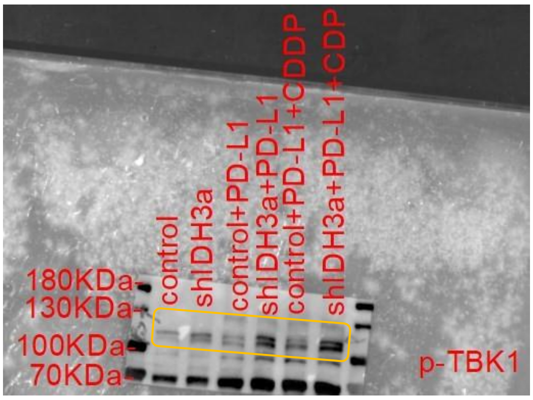

Fig 7I. p-IRF3

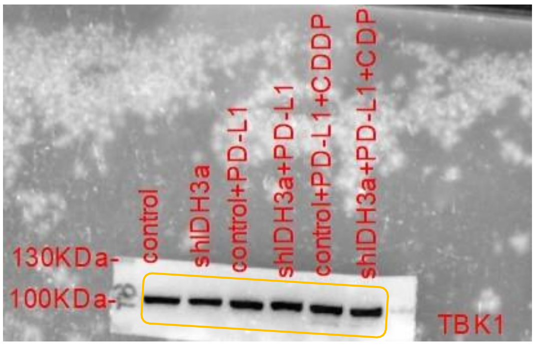

Fig 7I.IRF3

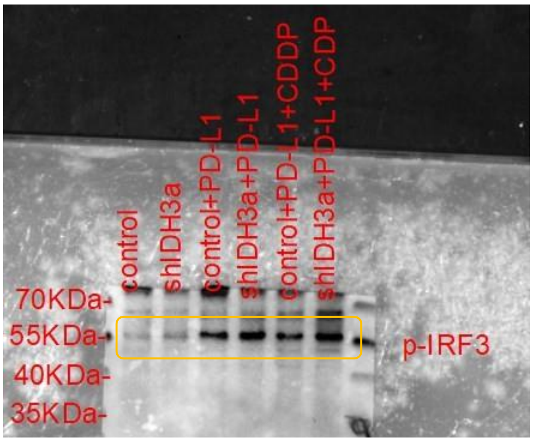

Fig 7I. p-p65

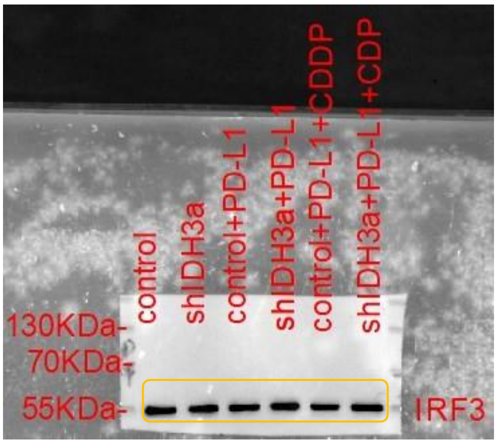

Fig 7I.p65

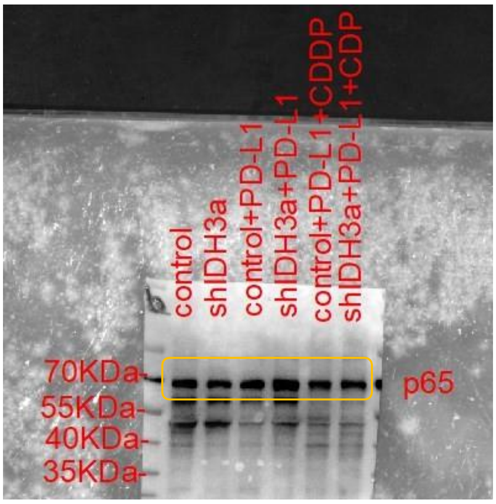

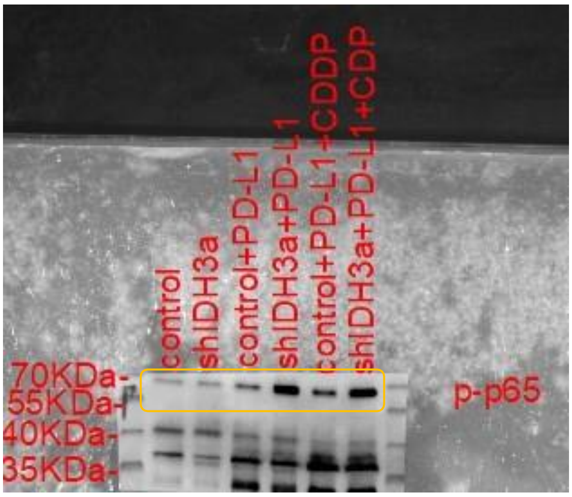

Fig 7I. β-tubulin

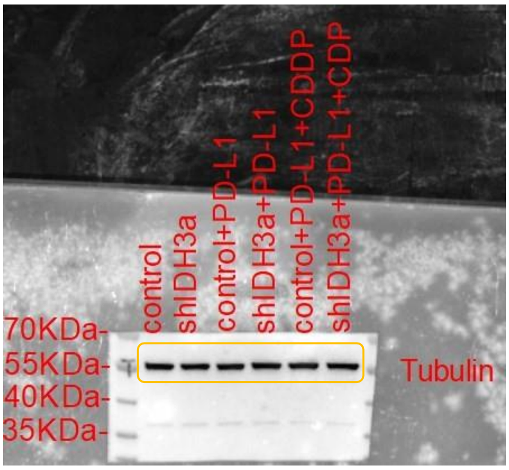

Original blots corresponding to Figure S3:

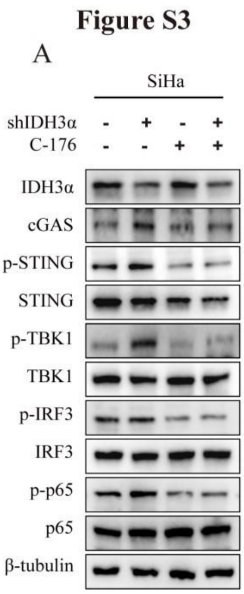

Fig S3A. SiHa IDH3A

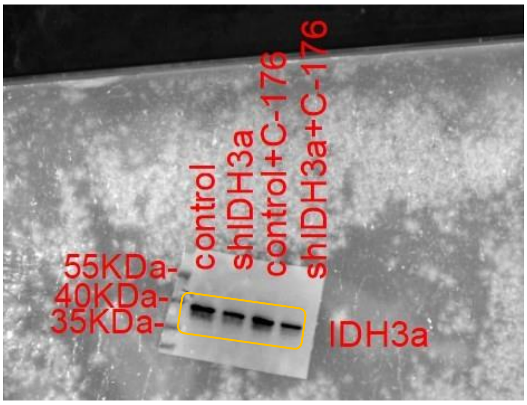

Fig S3A. SiHa cGAS

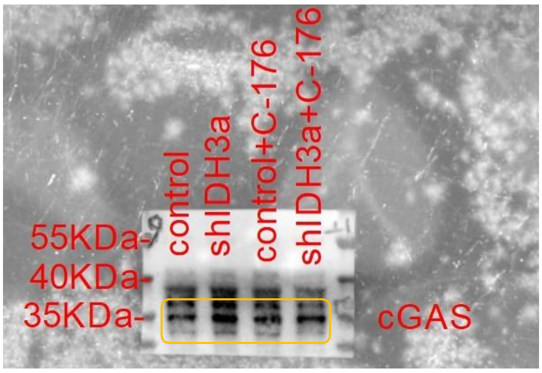

Fig S3A. SiHa p-STING

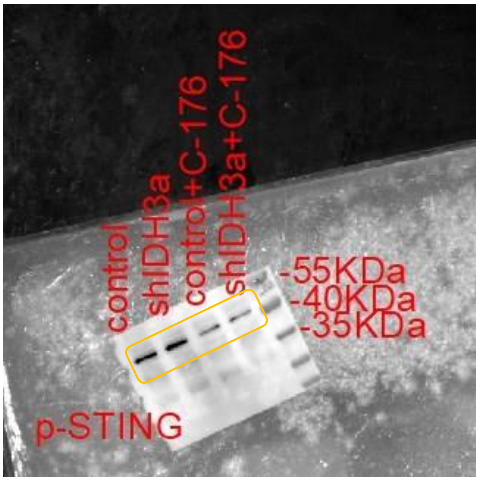

Fig S3A. SiHa STING

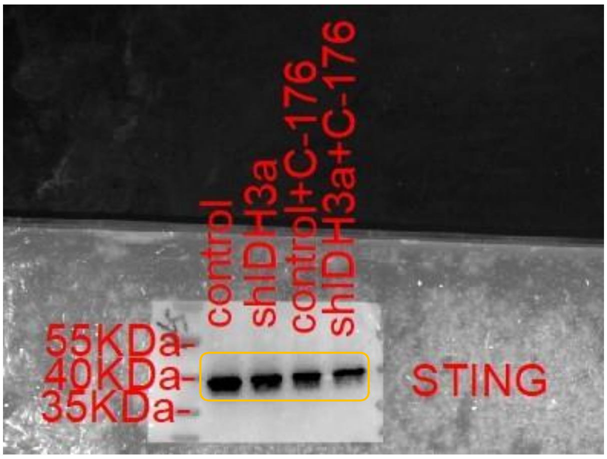

Fig S3A. SiHa p-TBK1

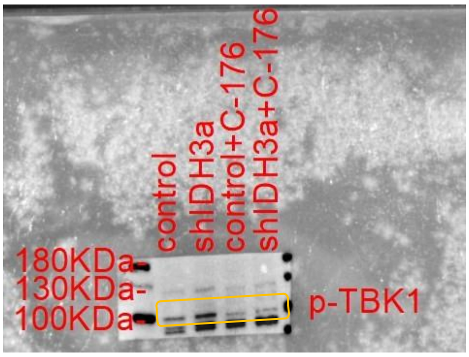

Fig S3A. SiHa TBK1

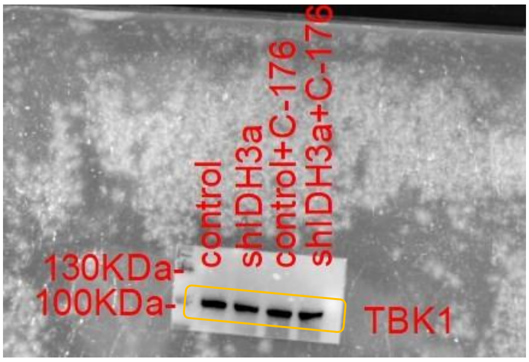

Fig S3A. SiHa p-IRF3

Fig S3A. SiHa IRF3

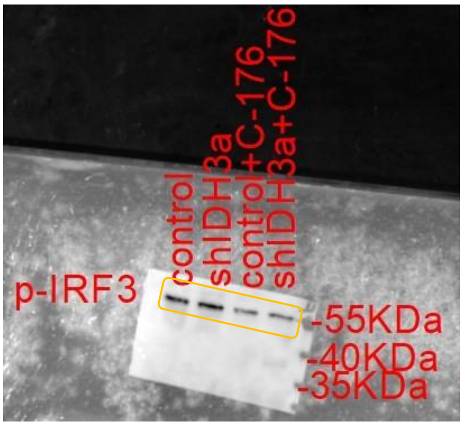

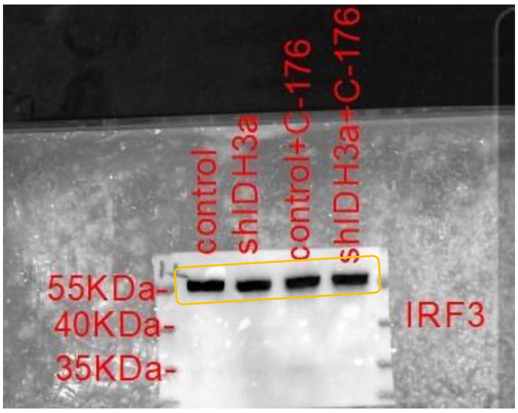

Fig S3A. SiHa p-p65

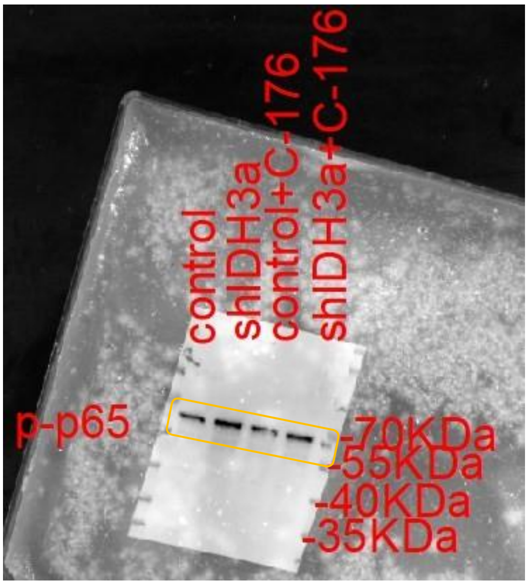

Fig S3A. SiHa p65

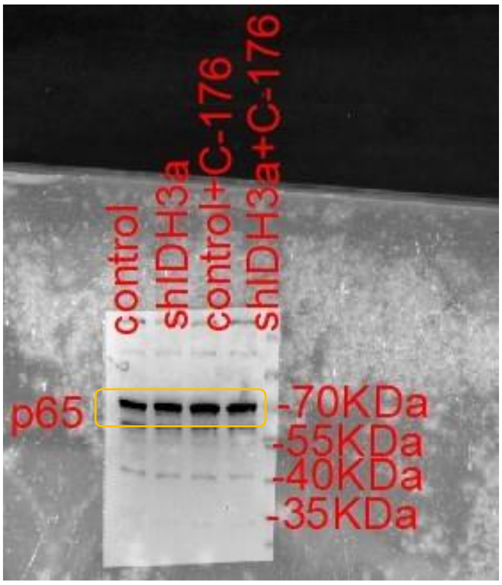

Fig S3A. SiHa β-tubulin

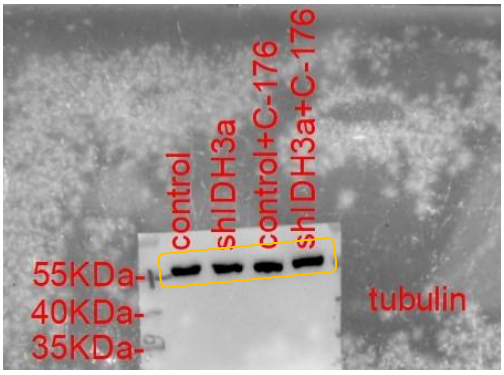

Original blots corresponding to Figure S4:

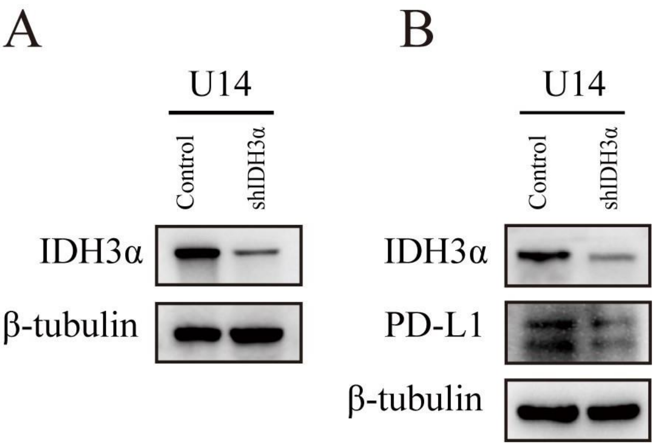

Fig S4A. U14 IDH3α

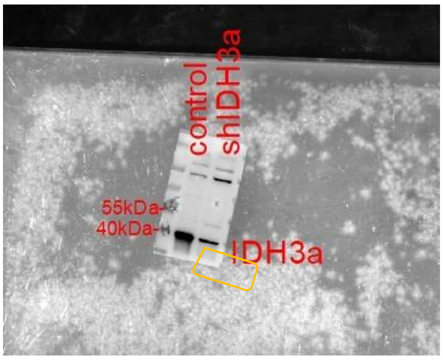

Fig S4A. U14 β-tubulin

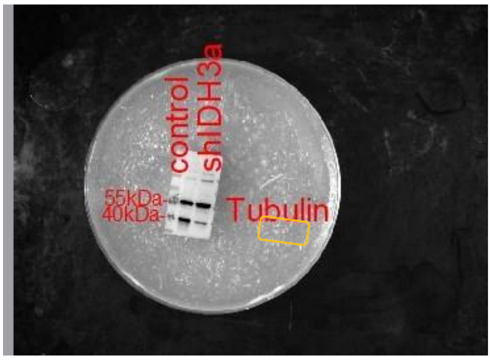

Fig S4B. U14 IDH3α

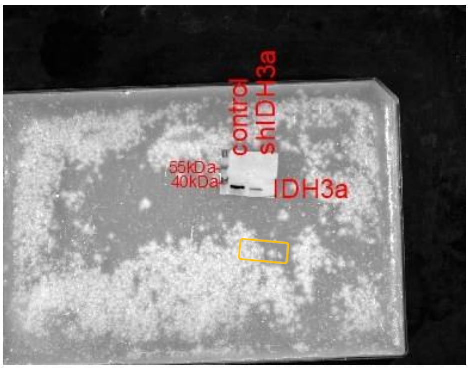

Fig S4B.U14 PD-L1

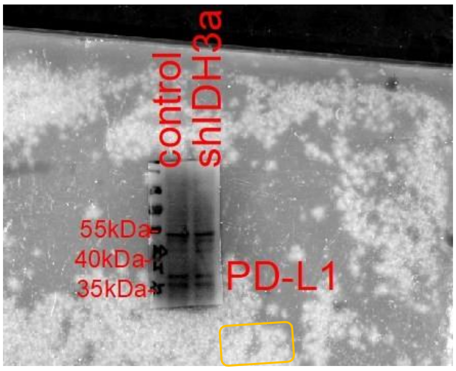

Fig S4B. U14 β-tubulin

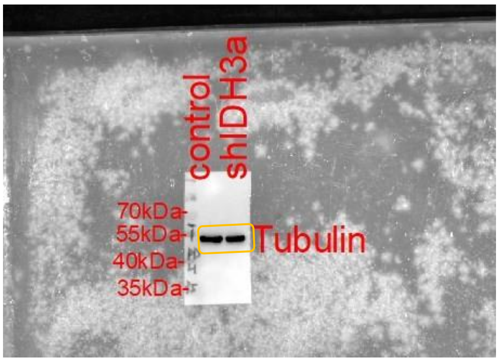

Figure S5: The uncropped Western blots.

Table S1. List of expression primers used in this study

Target	Forward primer	Reverse primer
**IDH3α(human)**	AATTTTTGAGTCGGTTCATGGG	TCAAGCTCTTTCCGTCCTTAAT
**IDH1 (human)**	CTATGATGGTGACGTGCAGTCG	CCTCTGCTTCTACTGTCTTGCC
**IDH2 (human)**	AGATGGCAGTGGTGTCAAGGAG	CTGGATGGCATACTGGAAGCAG
**E-cadherin (human)**	GCCTCCTGAAAAGAGAGTGGAAG	TGGCAGTGTCTCTCCAAATCCG
**N-cadherin (human)**	CCTCCAGAGTTTACTGCCATGAC	GTAGGATCTCCGCCACTGATTC
**Vimentin (human)**	AGGCAAAGCAGGAGTCCACTGA	ATCTGGCGTTCCAGGGACTCAT
**Slug (human)**	ATCTGCGGCAAGGCGTTTTCCA	GAGCCCTCAGATTTGACCTGTC
**LDHA (human)**	GGATCTCCAACATGGCAGCCTT	AGACGGCTTTCTCCCTCTTGCT
**HK2 (human)**	GAGTTTGACCTGGATGTGGTTGC	CCTCCATGTAGCAGGCATTGCT
**PKM2 (human)**	ATGGCTGACACATTCCTGGAGC	CCTTCAACGTCTCCACTGATCG
**CXCL1 (human)**	AGCTTGCCTCAATCCTGCATCC	TCCTTCAGGAACAGCCACCAGT
**CXCL2 (human)**	GGCAGAAAGCTTGTCTCAACCC	CTCCTTCAGGAACAGCCACCAA
**CCL5 (human)**	CCTGCTGCTTTGCCTACATTGC	ACACACTTGGCGGTTCTTTCGG
**CXCL10 (human)**	GGTGAGAAGAGATGTCTGAATCC	GTCCATCCTTGGAAGCACTGCA
**IFN-β (human)**	CTTGGATTCCTACAAAGAAGCAGC	TCCTCCTTCTGGAACTGCTGCA
**β-actin (human)**	CCTGGCACCCAGCACAAT	GGGCCGGACTCGTCATAC
**IDH3α(mouse)**	GCAGGACTGATTGGAGGTCTTG	GCCATGTCCTTGCCTGCAATGT
**CXCL1 (mouse)**	TCCAGAGCTTGAAGGTGTTGCC	AACCAAGGGAGCTTCAGGGTCA
**CXCL2 (mouse)**	CATCCAGAGCTTGAGTGTGACG	GGCTTCAGGGTCAAGGCAAACT
**CCL5 (mouse)**	CCTGCTGCTTTGCCTACCTCTC	ACACACTTGGCGGTTCCTTCGA
**CXCL10 (mouse)**	ATCATCCCTGCGAGCCTATCCT	GACCTTTTTTGGCTAAACGCTTTC
**β-actin (mouse)**	GTGCTATGTTGCTCTAGACTTCG	ATGCCACAGGATTCCATACC
